# Multi-omics integration highlights the role of ubiquitination in endometriosis fibrosis

**DOI:** 10.1186/s12967-024-05245-0

**Published:** 2024-05-12

**Authors:** Mengjie Yang, Hong Jiang, Xinyu Ding, Lu Zhang, Huaying Zhang, Jiahao Chen, Lijun Li, Xinqin He, Zhixiong Huang, Qionghua Chen

**Affiliations:** 1 Clinical Medical Research Center for Gynecological Reproductive Health of Fujian Province, Laboratory of Research and Diagnosis of Gynecological Diseases of Xiamen City, Department of Obstetrics and Gynecology, the First Affliated Hospital of Xiamen University, School of Medicine, Xiamen University, Xiamen, China; 2 National Institute for Data Science in Health and Medicine, Xiamen University, Xiamen, China; 3 Department of Obstetrics and Gynecology, The First Affiliated Hospital of Fujian Medical University, Fuzhou, China; 4 Reproductive Medicine Center, The First Affiliated Hospital of Fujian Medical University, Fuzhou, China

**Keywords:** Endometriosis, Proteomics, Ubiquitylomics, Fibrosis, TRIM33

## Abstract

**Background:**

Endometriosis, characterized by the presence of active endometrial-like tissues outside the uterus, causes symptoms like dysmenorrhea and infertility due to the fibrosis of endometrial cells, which involves excessive deposition of extracellular matrix (ECM) proteins. Ubiquitination, an important post-transcriptional modification, regulates various biological processes in human diseases. However, its role in the fibrosis process in endometriosis remains unclear.

**Methods:**

We employed multi-omics approaches on two cohorts of endometriosis patients with 39 samples. GO terms and KEGG pathways enrichment analyses were used to investigate the functional changes involved in endometriosis. Pearson’s correlation coefficient analysis was conducted to explore the relationship between global proteome and ubiquitylome in endometriosis. The protein expression levels of ubiquitin-, fibrosis-related proteins, and E3 ubiquitin-protein ligase TRIM33 were validated via Western blot. Transfecting human endometrial stroma cells (hESCs) with TRIM33 small interfering RNA (siRNA) in vitro to explore how TRIM33 affects fibrosis-related proteins.

**Results:**

Integration of proteomics and transcriptomics showed genes with concurrent change of both mRNA and protein level which involved in ECM production in ectopic endometria. Ubiquitylomics distinguished 1647 and 1698 ubiquitinated lysine sites in the ectopic (EC) group compared to the normal (NC) and eutopic (EU) groups, respectively. Further multi-omics integration highlighted the essential role of ubiquitination in key fibrosis regulators in endometriosis. Correlation analysis between proteome and ubiquitylome showed correlation coefficients of 0.32 and 0.36 for ubiquitinated fibrosis proteins in EC/NC and EC/EU groups, respectively, indicating positive regulation of fibrosis-related protein expression by ubiquitination in ectopic lesions. We identified ubiquitination in 41 pivotal proteins within the fibrosis-related pathway of endometriosis. Finally, the elevated expression of TGFBR1/α-SMA/FAP/FN1/Collagen1 proteins in EC tissues were validated across independent samples. More importantly, we demonstrated that both the mRNA and protein levels of TRIM33 were reduced in endometriotic tissues. Knockdown of TRIM33 promoted TGFBR1/p-SMAD2/α-SMA/FN1 protein expressions in hESCs but did not significantly affect Collagen1/FAP levels, suggesting its inhibitory effect on fibrosis in vitro.

**Conclusions:**

This study, employing multi-omics approaches, provides novel insights into endometriosis ubiquitination profiles and reveals aberrant expression of the E3 ubiquitin ligase TRIM33 in endometriotic tissues, emphasizing their critical involvement in fibrosis pathogenesis and potential therapeutic targets.

**Supplementary Information:**

The online version contains supplementary material available at 10.1186/s12967-024-05245-0.

## Introduction

Endometriosis, a prevalent and non-malignant gynecological condition, features active endometrial-like tissues outside the uterus, affecting organs like the ovaries, peritoneum, sacral ligaments, intestines, and other pelvic organs [1]. It affects 5–10% of females of reproductive age and causes dysmenorrhea, painful intercourse, infertility, and even pelvic malignant tumors [2]. Diagnosing endometriosis is challenging, requiring an invasive biopsy that may delay treatment [3]. Current treatments, including hormone therapy and surgery, have drawbacks like drug side effects and post-surgical ovarian dysfunction [4]. Fibrosis, characterized by abnormal extracellular matrix (ECM) protein accumulation, is pervasive in all endometriosis forms [5]. Ectopic lesions undergo dynamic processes (bleeding and repair) influenced by factors like immune cell infiltration, inflammatory cytokine release, and a heightened estrogen environment, contributing to excessive ECM buildup and fibrosis, leading to typical symptoms like dysmenorrhea and infertility [6]. Prior research indicates a positive link between dysmenorrhea severity and fibrosis extent in endometriosis. Moreover, anti-mullerian hormone (AMH) levels show a negative correlation with cortical fibrosis near endometriotic cysts [7]. The exact causes and processes underlying fibrosis in endometriosis are not well-defined, emphasizing the need to understand the molecular mechanisms associated with endometriosis and its fibrosis for effective early diagnosis and treatment strategies.

Recent technological advances have significantly enhanced the assessment of genome-wide gene activity. Current research aims to identify biomarkers and elucidate pathogenic mechanisms [8, 9]. Various specimens, including endometrial tissues [10, 11], blood [12], urine [13], peritoneal fluid [14], and follicular fluid [15], have undergone diverse omics analyses, providing valuable but predominantly single-omics-focused insights. Notably, a single gene often codes for multiple protein isoforms, each potentially serving distinct biological functions and subject to regulation by various post-translational modifications (PTMs) [16, 17]. Studies show poor correlation between protein and transcript levels [18, 19], a discrepancy also observed in tubal endometriosis [20], emphasizing the importance of proteomics, particularly modified-proteomics, in discovering novel biomarkers and targets.

Ubiquitination, a vital cellular process, attaches the conserved protein ubiquitin to a lysine residue on a substrate protein [21]. This attachment, facilitated by E1, E2, and E3 enzymes, involves activation, conjugation, and ligation steps, linking ubiquitin’s C-terminal glycine to the substrate [22, 23]. Ubiquitin comprises seven lysine residues (K6, K11, K27, K29, K33, K48, and K63), a methionine residue at the N-terminus (M1), and a glycine residue at the C-terminus (G76). Nine recognized ubiquitination modes, based on connection sites, regulate the activation, localization, and interactions of various cellular proteins, influencing cell signaling, stress response, DNA repair, and implicated in various human diseases [24]. Recent research highlights the essential association between ubiquitination and endometriosis pathogenesis [25]. It was reported that ubiquitin was expressed in endometriotic cells and was associated with the survival of ectopic stromal cells in endometriosis [26]. Irregularities in ubiquitin-modifying enzymes within ectopic lesions significantly influence endometriosis development. Importantly, ubiquitin-related pathways contribute to specific protein degradation, influencing cell proliferation, estrogen receptor expression, and other critical processes in endometriosis [27–35]. Despite literature outlining specific alterations in ubiquitination, substrates, or enzymes associated with endometriosis, a systematic analysis of the ubiquitination profile in endometriosis and its role in fibrosis is currently lacking.

In this study, we conducted a comprehensive investigation employing multi-omics approaches on two cohorts comprising 39 endometriosis patients. Proteomics identified 73,218 tryptic peptides and quantified 8032 unique proteins, revealing altered protein patterns, including dysregulation of ubiquitin-related enzymes. Integration of proteomics and transcriptomics unveiled genes implicated in ECM accumulation and impaired reproductive functions. Ubiquitylomics further delineated 8407 ubiquitinated lysine (Kub) peptides and 2678 Kub-proteins across tissues. Notably, multi-omics integration highlighted the role of ubiquitination in regulating fibrosis mediators. Further identification of ubiquitination in 41 crucial fibrosis-related proteins provides novel insights into molecular mechanisms and potential therapeutic targets. Finally, we uncovered the abnormal expression of E3 ubiquitin ligase TRIM33 in endometriotic tissues of endometriosis, along with its negative regulatory role in fibrosis of human endometrial stromal cells in vitro. This study, for the first time, provides a comprehensive understanding of endometriosis ubiquitination profiles and reveals the aberrant expression of the TRIM33 in endometriotic tissues, emphasizing their critical involvement in fibrosis pathogenesis and potential therapeutic targets.

## Methods

### Experimental design and statistical rationale

**Fig. 1:**
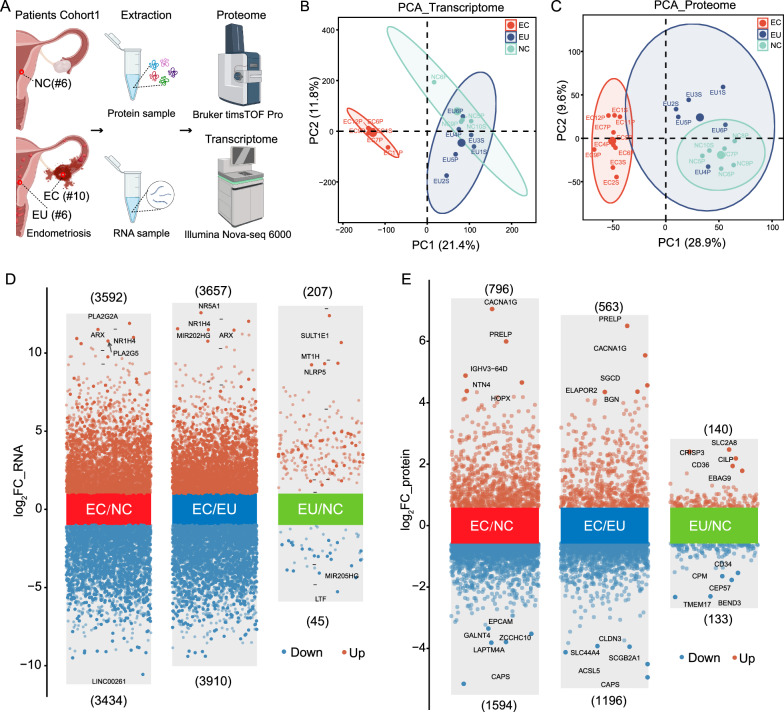
Overview of global transcriptomics and proteomics studies in endometriosis. **A** Schematic representation of the transcriptomic and proteomic strategies employed for the investigation of endometriosis; Schematic was created in BioRender.com; **B** PCA of transcriptomics data, illustrating the separation of EC, EU, and NC samples; **C** PCA of DIA-PASEF proteomic data, showing the differentiation between EC, EU, and NC samples; **D** Volcano plot depicting DEGs in EC versus NC, EC versus EU, and EU versus NC groups, with genes having an adjusted p-value < 0.05 and a FC > 2 considered as significantly DEGs; **E** Volcano plot displaying DEPs in EC versus NC, EC versus EU, and EU versus NC groups, with proteins having a p-value < 0.05 and a FC > 1.5 considered as significant DEPs. NC: normal endometria; EU: eutopic endometria; EC: ectopic endometria; DEGs: differentially expressed genes; DEPs: differentially expressed proteins; log2FC: log2Foldchange; PCA: principal component analysis; DIA-PASEF: parallel accumulation-serial fragmentation combined with data-independent acquisition

In this study, for Cohort 1, six control endometria (NC) from non-endometriosis patients, and six eutopic (EU) and 10 ectopic endometria (EC) from 10 ovarian endometriosis patients, underwent integrated transcriptomic and proteomic analyses using RNA-sequencing and parallel accumulation-serial fragmentation combined with data-independent acquisition (DIA-PASEF) strategy (Fig. 1A). In Cohort 2, label-free quantitative ubiquitylomics analysis was performed on five NC from non-endometriosis patients and paired samples of EU and EC endometria from six ovarian endometriosis patients (Fig. 4C). Additionally, samples from Cohort 3 were used for independent experimental validation. Samples from Cohorts 1 and 3 were collected at the Department of Obstetrics and Gynecology in the First Affiliated Hospital of Xiamen University, with ethical approval number KY2021-03 (Table S1). Cohort 2 samples were obtained from the Department of Obstetrics and Gynecology at the First Affiliated Hospital of Fujian Medical University (Ethical approval number: MRCTA, ECFAH of FMU [2021]484) (Table S1).

All patients had normal menstrual cycles and had not received hormonal therapy over the three months prior to surgery. Furthermore, all endometriosis patients were excluded from other gynecological diseases by B-ultrasonography and considering previous medical history. Control patients without a history of endometriosis were confirmed by ultrasonography. The EU and NC samples were acquired by hysteroscopy while the EC tissues were obtained by laparoscopy and confirmed by postoperative pathological analysis. All isolated specimens were washed twice with pre-cooled phosphate buffer saline and then frozen in liquid nitrogen for at least 30 min and stored at – 80 ℃.

For transcriptomic analyses, we conducted significance analysis using DEseq2 with Benjamini–Hochberg false discovery rate (FDR) correction; genes with an adjusted p < 0.05 and a fold change (FC) > 2 were deemed significantly differentially expressed genes (DEGs). In proteomic studies, significance analysis employed an unpaired t-test, and proteins with a p < 0.05 and a FC > 1.5 were regarded as significant differentially expressed proteins (DEPs). Ubiquitylomics studies involved significance analysis using an unpaired t-test, and proteins with a p < 0.05 and a FC > 1.5 were considered significant differentially ubiquitinated proteins (DUPs).

### RNA sequencing and quality control measures

Total RNA was extracted from endometrial tissues using TRIzol^®^ Reagent in accordance with the manufacturer's instructions (Magen Biotech, China). RNA samples were detected based on the A260/A280 absorbance ratio with a Nanodrop ND-2000 system (ThermoFisher Scientific, USA). The RNA integrity number (RIN) of RNA was determined by an Agilent Bioanalyzer 4150 system (Agilent Technologies, CA, USA). Only qualified samples were used for library construction.

Paired-end libraries were prepared using an ABclonal mRNA-seq Lib Prep Kit (ABclonal, China). mRNA was purified from 1 μg of total RNA using oligo (dT) magnetic beads followed by fragmentation using divalent cations at elevated temperatures in ABclonal First Strand Synthesis Reaction Buffer. Subsequently, first-strand cDNAs were synthesized with random hexamer primers and Reverse Transcriptase (RTs) using mRNA fragments as templates; this was followed by second-strand cDNA synthesis using DNA polymerase I, RTs, buffer, and dNTPs. The synthesized double-stranded cDNA fragments were then adapter-ligated to prepare the paired-end library. Adaptor-ligated cDNA was then used for polymerase chain reaction (PCR) amplification. PCR products were purified using the AMPure XP system (Beckman Coulter, Beverly, USA), and library quality was assessed on an Agilent Bioanalyzer 4150 system (Agilent Technologies, USA). Finally, sequencing was performed with an Illumina Novaseq 6000/MGISEQ-T7 instrument.

Raw data in FASTQ format were then processed. In this step, we removed the adapter sequence and filtered out low-quality reads (low quality, the number of lines with a string quality value less than or equal to 25 accounts for more than 60% of the entire reading) and N reads greater than 5% (N refers to the fact that base information could not be determined) to acquire clean reads that could be used for subsequent analysis. Then, clean reads were separately aligned to the reference genome with orientation mode using HISAT2 software (http://daehwankimlab.github.io/hisat2/) to obtain mapped reads. FeatureCounts (http://subread.sourceforge.net/) was then used to count the read numbers mapped to each gene. Then, the expected number of Fragments Per Kilobase of transcript sequence per Millions base pairs sequenced (FPKM) of each gene was calculated based on the length of each gene and the mapped read count.

### DIA-based quantitative proteomics analysis

#### Protein extraction and digestion

Endometrial tissue samples were collected and then SDT lysis buffer (4% SDS, 100 mM DTT, 150 mM Tris–HCl pH 8.0) was added. The samples were transferred into 2 mL centrifuge tubes pre-loaded with quartz sand and 1/4 inch ceramic beads (MP 6540–424). The samples were homogenized using an MP Fastprep-24 Automated Homogenizer (MP Biomedicals) (24 × 2, 6.0 M/S, 60 s, twice). Next, the samples were sonicated (2000W, 30 s on, 30 s off, for 10 cycles) and heated in a water bath for 8 min. The tubes were then centrifuged at 12500 g for 20 min, and the supernatant was carefully collected. The supernatant was filtered through a 0.22 µm filter membrane to obtain the filtrate. Protein quantification was carried out using the BCA Protein Assay Kit (Bio-Rad, USA). Equal aliquot from each sample in this experiment was pooled into one sample for DDA library generation and quality control.

Equal amounts of protein from each sample were added to SDT buffer (4% SDS, 100 mM dithiothreitol (DTT), 150 mM Tris–HCl pH 8.0). The detergents, DTT, and other low molecular weight components were then removed using urea buffer (UA, 8 M urea, 150 mM Tris–HCl pH 8.0) via repeated ultrafiltration (Microcon device, 10 kD). Subsequently, 100 μl of iodoacetamide (IAA) (100 mM IAA in UA buffer) was added to block reduced cysteine residues, and the samples were incubated in darkness for 30 min. The filters were washed three times with 100 μl of UA buffer and then twice with 100 μl of 25 mM NH_4_HCO_3_ buffer. Finally, the 100 ug protein were digested overnight at 37 °C with 4 μg of trypsin (Promega) in 40 μl of 25 mM NH_4_HCO_3_ buffer, and the resulting peptides were collected as filtrate. Added enough 20% trifluoroacetic acid (TFA) to the sample (at a 20:1 volume ratio), mixed well, and checked the pH using pH paper, ensuring it was below 3.

Digested pool peptides were then fractionated to 10 fractions using a Thermo Scientific™ Pierce™ High pH Reversed-Phase Peptide Fractionation Kit. Each fraction was concentrated by vacuum centrifugation and reconstituted in 15 µl of 0.1% (v/v) formic acid. Collected peptides were then desalted on C18 Cartridges (Empore™ SPE Cartridges C18 (standard density), bed I.D. 7 mm, volume 3 ml, Sigma) and reconstituted in 40 µl of 0.1% (v/v) formic acid. The iRT-Kits (Biognosys) peptides were spiked before DDA analysis.

#### DDA mass spectrometry assay and library generation

All fraction samples were analyzed by a hybrid trapped ion mobility spectrometry quadrupole time-of-flight mass spectrometer (TIMS-TOF Pro, Bruker Daltonics, Bremen, Germany) with Evosep One System liquid chromatography (Evosep, Denmark), with a gradient of 15SPD. The MS was operated in the data-dependent mode for ion mobility-enhanced spectral library generation. We set the accumulation and ramp time to 100 ms each and recorded mass spectra in the range from *m/z* 100–1700 in positive electrospray mode; dynamic exclusion was 24.0 s. The ion source voltage was set to 1500 V, the temperature was set to 180℃, and dry gas was set to 3L/min. Ion mobility was scanned from 0.6 to 1.6 Vs/cm^2^; then, eight cycles of PASEF MS/MS scans were performed. The representative spectrum was added to the library, best scoring was the criteria for choice, and all identified peptides were used to generate the library. Each precursor ion retained the top 3–6 fragment macro-ions.

The FASTA sequence database was searched for DDA library data with Spectronaut™ 14.4.200727.47784 (Biognosys) software. The database was downloaded from http://www.uniprot.org. IRT peptide sequences were then added (Biognosys |iRT Kit|). The parameters were set as follows: the enzyme was set to trypsin, the max missed cleavages were set to 2, fixed modification was set to carbamidomethyl (C), and dynamic modification was set to oxidation (M) and acetyl (Protein N-term). All reported data were based on 99% confidence for protein identification as determined by false discovery rate (FDR = N (decoy)*2/(N(decoy) + N(target))) < 1%. Finally, a DDA library was built containing 125,185 tryptic peptides and 11,269 protein groups.

#### DIA mass spectrometry assay and data analysis

Next, 200 ng of peptide from each sample was mixed with an appropriate amount of iRT standard peptide. The peptides from each sample were then analyzed by nano LC–MS/MS operating in the DIA mode, with a gradient of 15SPD. The mass spectrometer collected ion mobility MS spectra over a mass range of *m/z* 100–1700. We defined up to eight windows for single 100 ms TIMS scans according to the *m/z*-ion mobility plane to acquire MS2 data. During PASEF MSMS scanning, the collision energy was ramped linearly as a function of the mobility from 20 eV at 1/K0 = 0.6 Vs/cm^2^ to 59 eV at 1/K0 = 1.6 Vs/cm^2^.

DIA data were analyzed with Spectronaut™ 14.4.200727.47784 (Biognosys), by searching the constructed library. The main software parameters were as follows: retention time prediction type was set to dynamic iRT, interference on MS2 level correction was enabled, and cross-run normalization was enabled. The MS mass tolerance was set to dynamic, meaning that Spectronaut computed the optimal mass tolerances for data extraction and scoring based on extensive mass calibration. All results were filtered based on a Q value cutoff of 0.01 (equivalent to an FDR < 1%).

### Label free quantitative ubiquitylomics analysis

#### Protein extraction and digestion

Sample lysis and protein extraction used a urea buffer (UA, 8 M Urea, 100 mM Tris/HCl, pH 8.5). Protein quantification employed the Bradford Protein Assay Kit. Each sample, consisting of 20 µg of protein, was mixed with 5X loading buffer and boiled for 5 min. Proteins were separated on a 12.5% SDS-PAGE gel (constant current 14 mA, 90 min) and visualized with Coomassie Blue R-250 staining. DTT (final concentration 10 mM) was added to each sample, mixed at 600 rpm for 1.5 h (37 ℃), then cooled to room temperature. IAA (final concentration 50 mM) was added to the mixture and incubated in the dark for 30 min. UA concentration was diluted to 2 M with a fourfold volume of 50 mM Tris HCl (pH 8.0). Trypsin was added to the samples at a trypsin: protein (wt/wt) ratio of 1:50 and incubated at 37 ℃ for 15–18 h (overnight). Following digestion, TFA (final concentration 0.1%) was added, and the pH was adjusted to pH ≤ 3 with 10% TFA. Digested peptides from each sample were desalted using C18 Cartridges (Empore™ SPE Cartridges C18, standard density, bed I.D. 7 mm, volume 3 ml, Sigma) and lyophilized for further use.

#### Ubiquitinated peptides enrichment

The endometrial samples were reconstituted in 1.4 mL of pre-cooled immunoaffinity purification (IAP) buffer. Pretreated Anti-K-ε-GG antibody beads (PTMScan Ubiquitin Remnant Motif (K-ε-GG) Kit, Cell Signal Technology) were added, followed by incubation at 4 °C for 1.5 h. The mixture was then centrifuged at 2000 ×*g* for 30 s, and the supernatant was discarded. The Anti-K-ε-GG antibody beads were washed three times with 1 mL pre-cooled IAP Buffer and then three times with pre-cooled water. Subsequently, 40 μL of 0.15% TFA was added to the washed beads, incubated for 10 min at room temperature, followed by the addition of 0.15% TFA again. The mixture was centrifuged at 2,000 × g for 30 s, and the supernatant was desalted using C18 STAGE Tips.

### Identification and quantitation of modified proteins

LC–MS/MS analysis was performed using a timsTOF Pro mass spectrometer (Bruker) coupled to Nanoelute (Bruker Daltonics) for 120 min. Peptides were loaded onto a homemade C18-reversed phase analytical column (Thermo scientific EASY column, length 25 cm, I.D. 75 μm, particle size 1.9 μm) in buffer A (0.1% Formic acid) and separated with a linear gradient of buffer B (99.9% acetonitrile and 0.1% Formic acid) at a flow rate of 300 nL/min. The gradient elution program proceeds as follows: from 0 to 3 min, Buffer B is linearly increased from 2 to 8%; from 3 to 110 min, Buffer B is linearly increased from 8 to 40%; from 110 to 115 min, Buffer B is linearly increased from 40 to 90%; and from 115 to 120 min, Buffer B is maintained at 90%. The mass spectrometer operated in positive ion mode, collecting ion mobility MS spectra in the mass range of m/z 100–1700 and 1/k0 of 0.6 to 1.6. Subsequently, 10 cycles of PASEF MS/MS were performed with a target intensity of 1.5 k and a threshold of 2500. Active exclusion was enabled with a release time of 0.4 min. MS raw data for each sample were combined and analyzed for identification and quantitation using MaxQuant software.

### Bioinformatic analysis

The normalized omics data were imported into SIMCA-P (version 14.1, Umetrics, Umea, Sweden) and subjected to multivariate data analysis. The Euclidean distance algorithm for similarity measure and average linkage clustering (clustering uses centroids of observations) were selected for hierarchical clustering. Principal component analysis (PCA) was conducted using the "FactoMineR" (http://cran.r-project.org/web/packages/FactoMineR/index.html) and "factoextra" (https://CRAN.R-project.org/package=factoextra) packages in R.

Gene ontology (GO) terms were mapped and sequences were annotated using program Blast2GO software (https://www.biobam.com/download-blast2go/). Following annotation steps, DEGs, DEPs and DUPs were blasted against the online Kyoto Encyclopedia of Genes and Genomes (KEGG) database (http://geneontology.org/) to retrieve KEGG orthology data and were subsequently mapped to pathways in KEGG. Enrichment analysis was applied based on Fisher’s exact test using the "ClusterProfiler" package in R while considering the entire set of DEGs, DEPs and DUPs as background datasets. Furthermore, only functional categories and pathways with p-values under a threshold of 0.05 were considered significant. GO term clusters were analyzed according to term similarity using the "simplifyEnrichment" package (https://github.com/jokergoo/simplifyEnrichment) in R.

In addition, CELLO (http://cello.life.nctu.edu.tw/), a multi-class SVM classification system, was used to predict protein subcellular localization.

### Quantitative real-time polymerase chain reaction (qRT-PCR) analysis

qRT-PCR analysis was performed as described in our previous work [36]. The primers used in the experiments were synthesized by Sangon Biotech (Shanghai, China). The detailed primer sequences were as follows (5′-3′): TRIM33, forward CCGGCAGGTGAAGCATGTTAT and reverse GCTTGCTGTATAGTAGTGCTGTG; GAPDH, forward GGAGCGAGATCCCTCCAAAAT and reverse GGCTGTTGTCATACTTCTCATGG.

### Western Blot

Protein samples from endometrial tissues and cells were exacted with RIPA lysis buffer (Abcam, Cambridge, UK) with protease and phosphatase inhibitors. Extracted proteins were then mixed with SDS-PAGE loading buffer, separated by 10% SDS-PAGE and transferred onto PVDF membranes (#03010040001; Roche, Basel, Switzerland). The membranes were incubated with primary antibodies overnight at 4 °C and then immunoblotted with a corresponding HRP-labeled secondary antibodies for 1 h at room temperature. The information and dilution rates of antibodies in this study were Ubiquitin (abcam, #ab134953, 1:1000), Ubiquitin (linkage-specific K63) (abcam, #ab179434, 1:1000), Ubiquitin (linkage-specific K48) (abcam, #ab140601, 1:1000), α-SMA (Proteintech, #67,735–1-Ig, 1:20,000), FN1 (Proteintech, #66,042–1-Ig, 1:2000), Collagen1 (Proteintech, #67,288–1-Ig, 1:5000), FAP (CST, #52,818, 1:1000), SMAD2 (CST, #5339, 1:1000), TGFBR1 (abcam, ab235578, 1:1000), TRIM33 (abcam, ab300146, 1:1000), p-SMAD2 (abcam, ab188334, 1:1000), and GAPDH (Proteintech, #60,004–1-Ig, 1:10,000). Finally, signals were visualized by enhanced chemiluminescence. Blots were scanned and analyzed with a ChemiDoc MP Imaging System (Bio-Rad; California, USA).

### Cell culture

Human endometrial stromal cells (hESCs) were purchased from the American Type Culture Collection (ATCC) and cultured within DMEM/F12 (phenol red −/−) (BasalMedia, Shanghai, China) containing 10% carbon adsorption FBS and 1% penicillin–streptomycin (Gibco, Waltham, MA, USA) in dishes at a 10% CO_2_ cell incubator at 37 ℃.

### Small interfering RNA (siRNA) and transfection

All the siRNAs were purchased from RiboBio. The negative control siRNA (#1,027,281) was used as siRNA control. Sequences for target siRNA was TRIM33, 5′-GCAGCCUUGUUAAUGGAAAGTT-3′. Following the manufacturer's protocols, transfection was achieved using Lipofectamine RNAiMAX (Invitrogen) for siRNA.

## Results

### Transcriptomic and proteomic atlas in endometriosis

We acquired 61,579 transcripts (corresponding to 39,412 mRNAs) at the transcriptome level by RNA sequencing. The transcriptomic data showed clear stratification among different groups according to PCA analysis (Fig. 1B). mRNA expression was more heterogeneous in the EC group in which 7026 mRNAs were differentially regulated (3592 up-regulated and 3434 down-regulated) when compared to the NC group; 7567 mRNAs were differentially regulated (3657 up-regulated and 3910 down-regulated) when compared with the EU group (Fig. 1D). However, compared with the NC group, only 252 mRNAs differed significantly (207 up-regulated and 45 down-regulated) in the EU group. The aforementioned results suggest dysregulation in the gene expression profile within endometriotic tissue, with notable heterogeneity observed when comparing EC tissue to EU and NC endometrium.

Next, we performed GO and KEGG enrichment analyses using the DEGs identified (Table S2). The upregulated mRNAs in the EC group enriched cell adhesion, migration, proliferation, cell communication, inflammatory response, regulation of immune system process, angiogenesis, and collagen-containing extracellular matrix (ECM) (Fig. S1A). The pathways enriched following the up-regulated mRNAs included the complement and coagulation cascades, cytokine-cytokine receptor, calcium, and the PI3K-AKT, MAPK, Rap1, Ras, JAK-SAT, TGF-β, and TNF signaling pathways (Fig. S1C, D). In contrast, the down-regulated mRNAs in the EC group showed enrichment in ciliary motility, internal dynein arm assembly, and reproductive processes, with a significant decrease in their levels (Fig. S1B) and were associated with homologous recombination, the cell cycle, p53 and various metabolic pathways (Fig. S1C, D). There was less significant enrichment of the biological pathways in the EU group when compared with the NC group, among which ECM-receptor interaction, phagosome, and histidine metabolism were enhanced; in contrast, there was less enrichment in the P53 signaling pathway, cytochrome P450 response to xenobiotics metabolism, and the biosynthesis of steroid hormones (Fig. S1E). Therefore, the transcriptome exhibited a significant change in biological function in terms of ectopic lesions, and there was little difference between eutopic and normal endometria.

Advanced mass spectrometry technology (DIA-PASEF) was applied to delineate the proteomic landscape. Quality control was performed to ensure the quality of the proteomic data (Fig. 1C). Using a customized analytical strategy, we identified 73,218 tryptic peptides, distinguishing unique 8032 proteins across all tissues in each group. Compared with the NC and EU groups, respectively, there were 2390 and 1759 proteins that showed significant changes in the EC group (Fig. 1D). Of these, 796 were up-regulated and 1594 were down-regulated in the EC group when compared with the NC group, while 563 were up-regulated and 1196 were down-regulated when compared to the EU group. Remarkably, the number of down-regulated proteins was approximately two-fold higher than the number of up-regulated proteins in the EC group. Similar to the transcriptomic results, the number of DEPs in the EU/NC group was lower than that in the EC/NC and EC/EU groups, among which 140 were up-regulated and 133 were down-regulated (Fig. 1D). This finding was consistent with previous transcriptomic and proteomic reports in endometriosis. The small differences between the NC and EU groups implied that the implantation of endometria in ectopic locations led to more significant changes in gene expression.

**Fig. 2:**
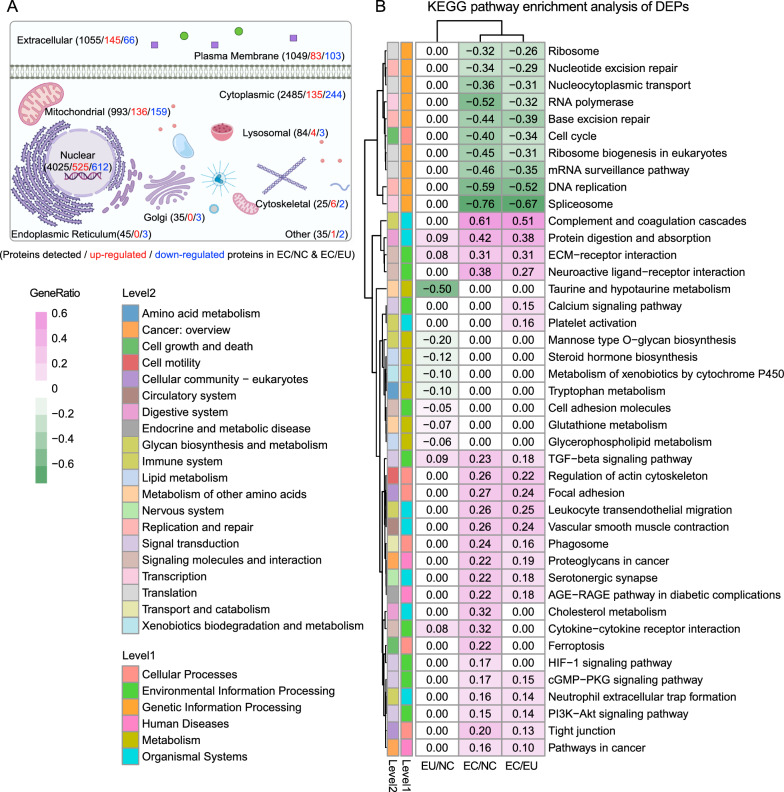
Functional enrichment analysis of proteomics data in endometriosis. **A** Subcellular distribution of all identified proteins, as well as those up-regulated and downregulated, was determined in the EC versus NC and EC versus EU groups. **B** KEGG pathways enrichment analysis of DEPs in EC versus NC, EC versus EU, and EU versus NC groups. Heat maps display enrichment factors, highlighting pathways with at least one significant comparison. Numbers denote Gene Ratio, with black indicating statistically significant enrichments (p-value < 0.05) and gray indicating non-statistically significant enrichments. The background is shaded in light purple for up-regulated pathways and light green for down-regulated pathways. DEPs: differentially expressed proteins; GO: Gene Ontology; KEGG: Kyoto Encyclopedia of Genes and Genomes

Next, the subcellular distribution of the DEPs both in the EC/NC and EC/EU groups were analyzed; most of the DEPs were distributed in the nucleus, cytoplasmic, mitochondrial, extracellular matrix, and plasma membrane (Fig. 2A). Interestingly, there were roughly twice as many upregulated proteins in the ECM and cytoskeleton compared to downregulated proteins, while more downregulated proteins were found in other organelles. This indicated notable activation changes in ECM and cytoskeleton-related functions in endometriotic tissue. The hypothesis was further confirmed by additional functional enrichment analysis of DEPs, which showed that up-regulated proteins in the EC group were implicated in protein activation, immune response, inflammatory response, extracellular structure organization and ubiquitin ligase complex (Fig. S2A, Table S3). KEGG pathway analysis also revealed the significant enrichment of complement and coagulation cascades, protein digestion and absorption, ECM-receptor interaction, vascular smooth muscle contraction, calcium, the PI3K-AKT pathway and the TGF-β signaling pathway in the EC group (Fig. 2B) (Table S3); all these analyses were highly consistent with the transcriptomic results (Fig. S1). However, RNA splicing, RNA processing, ribosome biogenesis, RNA binding, nucleic acid metabolic processes (Fig. S2B), as well as spliceosome, mRNA surveillance pathway, ribosome biogenesis in eukaryotes, DNA replication, nucleocytoplasmic transport, RNA degradation, and mismatch repair pathways (Fig. 2B), were significantly enriched among the down-regulated proteins in the EC group.

To demonstrate the authenticity of our data, we compared our proteomic results with previous mass spectrometry datasets published since 2010 (Fig. S2C). As shown in Fig. S2D, the proteomic data reported by Vehmas [37] included 214 DEPs, of which 213 proteins were also present in our dataset; a total of 164 mutual DEPs were identified. The data reported by Irungu [38] identified 1581 proteins, of which 704 were defined as DEPs (Fig. S2E); 1349 of these proteins were also present in our dataset, and 257 overlapping DEPs were identified. Furthermore, 941 of the 1349 proteins retained a consistent trend for change as described in our proteomic data. Hence, our proteomic findings demonstrated a strong alignment with datasets from prior studies, with our sequencing depth being notably deeper, thereby unveiling a more comprehensive array of information.

In summary, our study systematically uncovered the dysregulation of mRNA and protein expression and function in endometriosis. There was notable heterogeneity in gene expression within ectopic tissues compared to normal and eutopic endometrium. We identified a series of upregulated mRNA and proteins linked to inflammation and extracellular matrix response, with significant enrichment in associated functions and pathways. Furthermore, at the proteomics level, we observed downregulation of proteins in the EC group, predominantly implicated in fundamental cellular processes like RNA splicing and ribosome biogenesis, which have been less explored in previous research.

### Integrated transcriptomics and proteomics analysis in endometriosis

**Fig. 3:**
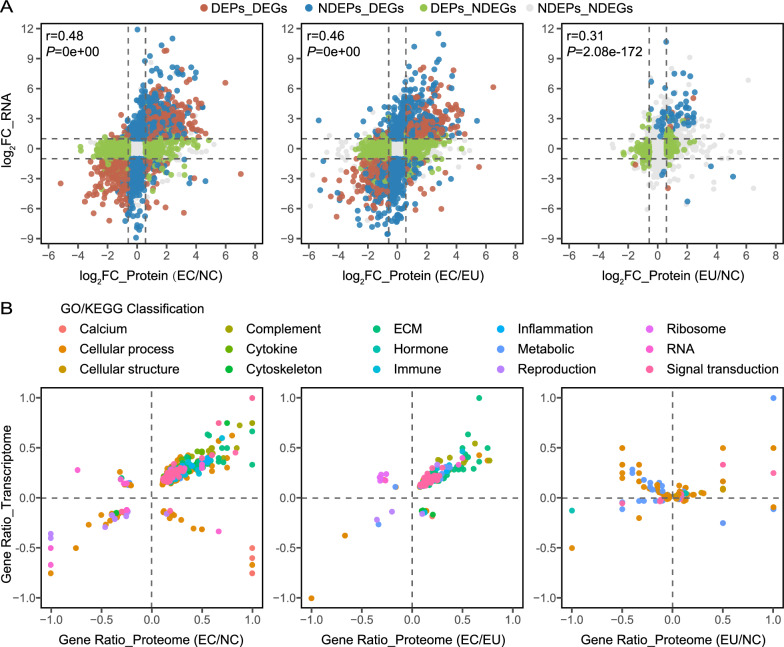
Integrated transcriptomics and proteomics analysis in endometriosis. **A** Scatterplot illustrating the relationship between changes in protein and mRNA abundances. The log_2_FC in mRNA abundance is plotted against the log_2_FC in protein abundance. The scatterplot is divided into sectors using a two-fold cutoff (adjusted p-value < 0.05 and |log_2_FC|> 1 for mRNA; p-value < 0.05 and |log_2_FC|> 0.58 for protein); **B** The scatterplot displays the results of GO and KEGG annotation enrichment analysis based on DEPs and DEGs that were significantly dysregulated at both protein and mRNA levels. Different types of enrichment annotation classifications are color-coded as explained in the legend. DEGs: differentially expressed genes; DEPs: differentially expressed proteins; NDEPs: non-DEPs; NDEGs: non-DEGs; log_2_FC: log_2_Foldchange

It was possible to identify the relationships between mRNAs and proteins with our data because the sequencing was performed using the same tissue specimens from patient cohorts. The distribution of genes was subdivided with a scatterplot to identify the genes associated with alterations between mRNA and protein levels (using a two-fold cutoff: adjusted p < 0.05 and |log_2_FC|> 1 for mRNA; p < 0.05 and |log_2_FC|> 0.58 for protein) (Table S4). As shown in Fig. 3A**,** when comparing EC and NC groups, the intersection of the DEPs and non-DEGs (NDEGs) (green) reached 21.3% (1703) of the total number of genes; the section including NDEPs non-DEPs (NDEPs) and DEGs (blue) was 11.0% (876) of the total number of genes; only 8.3% (665) of genes showed significant changes at both the mRNA and protein level (red). There were 7982 genes detected in the intersection of the EC and EU group (r = 0.46, p < 0.05), the proportion of DEPs and DEGs was 6.2% (498); the proportion of DEPs and NDEGs were 15.7% (1255) while the proportion of NDEPs and DEGs was 12.7% (1017). However, the overlap between the EU and NC groups containing 7975 matched genes showed only small differences in the expression of mRNA and protein. It was notable that we found a large number of genes exhibiting changes in different directions and opposing mRNA and protein levels, thus suggesting that there may be significant changes in post-transcriptional regulation and post-translational modification in endometriosis.

Next, we performed a further combined analysis of functional enrichment according to transcriptomic and proteomic data. As shown in Fig. 3B, the terms related to complement, cytokine, cytoskeleton, ECM, inflammation, and signal transduction showed significantly up-regulated changes at both the transcriptomics and proteomics level in the EC group. These results aligned with the functional enrichment analysis results from the mRNA and protein (DEPs and DEGs) in the EC group, which involved the activation of membrane attack complex, inflammatory response, ECM-receptor interaction, and the complement and coagulation cascades (Fig. S3). The reproductive terms were significantly enriched by the down-regulated genes in the EC group. These results supported the presence of inflammation and fibrosis in the endometriotic lesions, accompanied by reproductive functional deficiencies.

### Ubiquitination profiling landscape in endometriosis

**Fig. 4:**
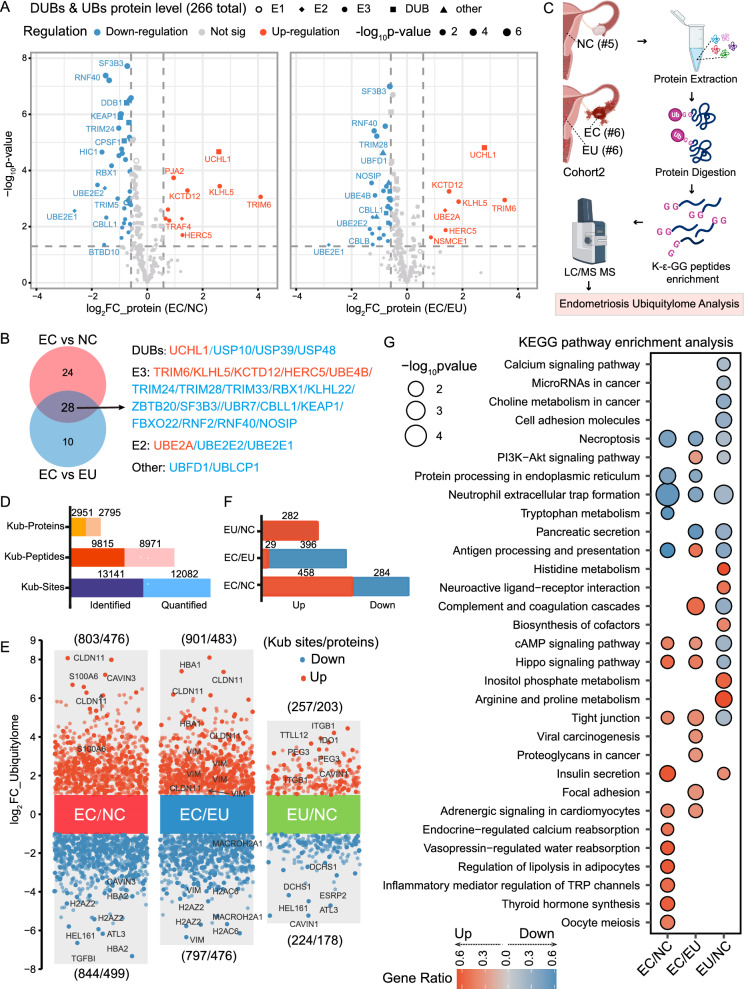
Ubiquitination profiling landscape in endometriosis. **A** Volcano plot displaying DUBs and UBs in EC versus NC, and EC versus EU groups, with proteins having a p-value < 0.05 and a FC > 1.5 considered as significant DEPs; the shape of the points represented the classification of ubiquitin-related enzymes;** B** The Venn diagram showing 28 enzymes that were dysregulated in both the EC/NC and EC/EU groups; Enzymes in red font were upregulated, while those in blue font were downregulated. **C** Schematic representation of the ubiquitylomics strategy employed for the investigation of endometriosis; Schematic was created in BioRender.com; **D** A glance at statistics for identification and quantification in ubiquitylomics data;** E** Volcano plot depicting differentially expressed Kub-sites and corresponding proteins in EC versus NC, EC versus EU, and EU versus NC groups, with sites having an p-value < 0.05 and a FC > 2 considered as significantly Kub-sites. **F** A glance at statistics for Kub-sites identified exclusively in one group; **G** Bubble charts illustrating the enrichment analysis of KEGG pathways of DUPs in EC versus NC, EC versus EU, and EU versus NC groups, respectively. The size of each bubble indicates the Gene Ratio and color represents the − log_10_(p-value) of enrichment. NC: normal endometria; EU: eutopic endometria; EC: ectopic endometria; UBs: ubiquitinating modifying enzymes; DUBs: deubiquitinases; Kub: ubiquitinated lysine; DUPs: differentially ubiquitinated proteins

Previous research indicates that ubiquitination plays a role in endometriosis pathogenesis by regulating protein degradation. Here, we used proteomic data to systematically analyze changes in the expression of ubiquitinating modifying enzymes (UBs) and deubiquitinases (DUBs) in endometriosis. We identified 266 DUBs and UBs across all groups (Fig. 4A) (Table S5). Compared to the NC and EU groups, 52 and 38 enzymes, respectively, showed significant changes in the EC group, with 28 common enzymes identified between the two comparison groups (Fig. 4B). Among these, 10 were up-regulated and 42 were down-regulated in the EC group compared to the NC group, while 8 were up-regulated and 30 were down-regulated compared to the EU group. This dysregulation of DUBs and UBs in the EC group suggests abnormal ubiquitination profiles in ectopic endometrial tissues.

To further explore the role of ubiquitination in regulating protein expression and identify new molecular mechanisms in endometriosis, we analyzed ubiquitin profiles in an independent cohort of 17 samples (Fig. 4C). Ubiquitylome data revealed stratification of the EC group from the EU and NC groups according to PCA analysis (Fig. S4A). We identified 2951 Kub-proteins, 9815 Kub-peptides, and 13,141 Kub-sites through ubiquitylome analysis. Among these, 8971 Kub-peptides and 12,082 Kub-sites were quantifiable on 2795 Kub-proteins (Fig. 4D). Venn analysis revealed 8407 Kub-peptides, identifying 2678 unique Kub-proteins across all tissues in each group (Fig. S4B). Of these, 803 sites in 476 proteins were up-regulated and 844 sites in 499 proteins were down-regulated in the EC group compared to the NC group, while 901 sites in 483 proteins were up-regulated and 797 sites in 476 proteins were down-regulated compared to the EU group **(**Fig. 4E**)**. Compared to the NC group, only 481 Kub-sites differed significantly (257 up-regulated and 203 down-regulated) in the EU group. We also analyzed sites that were ubiquitinated only in each group, as shown in Fig. 4F. Subcellular localization analysis of differentially ubiquitinated proteins (DUPs) indicated that they were mainly located in the nucleus, cytoplasm, plasma membrane, and extracellular matrix (Fig. S4C, D). The heatmap displayed the top 20 differentially Kub-sites and corresponding proteins in the EC group compared to the EU and NC groups (Fig. S4E). The above findings suggested aberrant ubiquitination modification of proteins in endometriosis, potentially implicating its role in the onset or progression of the disease.

Thus, to evaluate the impact of ubiquitination on endometriosis physiological processes, we conducted GO and KEGG enrichment analysis (Table S6). Identified DUPs in the EC group were enriched in GO terms including platelet aggregation, nucleosome, cell–cell adhesion, cytoskeleton, contractile fiber, and protein-DNA complex (Fig. S4F). Up-regulated DUP-associated pathways included tight junction, focal adhesion, cAMP, PI3K-Akt, and hippo signaling pathways (Fig. 4G). Conversely, down-regulated DUPs were enriched in necroptosis, protein processing in endoplasmic reticulum, and neutrophil extracellular trap formation.

### The relationship between global proteome and ubiquitylome in endometriosis

Pearson’s correlation coefficient analysis was conducted between the global proteome and ubiquitylome in endometriosis, revealing correlation coefficients of 0.22 and 0.32 in the EC/NC and EC/EU groups, respectively, while the EU/NC group showed a coefficient of -0.17 (Fig. S5A). Furthermore, correlation coefficients for differentially ubiquitinated proteins (DUPs) were calculated, resulting in coefficients of 0.42 (EC/NC), 0.52 (EC/EU), and − 0.32 (EU/NC), respectively (Fig. S5B). This indicated a positive correlation between the global proteome and ubiquitylome in the EC group compared with the NC and EU groups, suggesting consistent changing patterns in protein expression and ubiquitination in ectopic tissues.

**Fig. 5:**
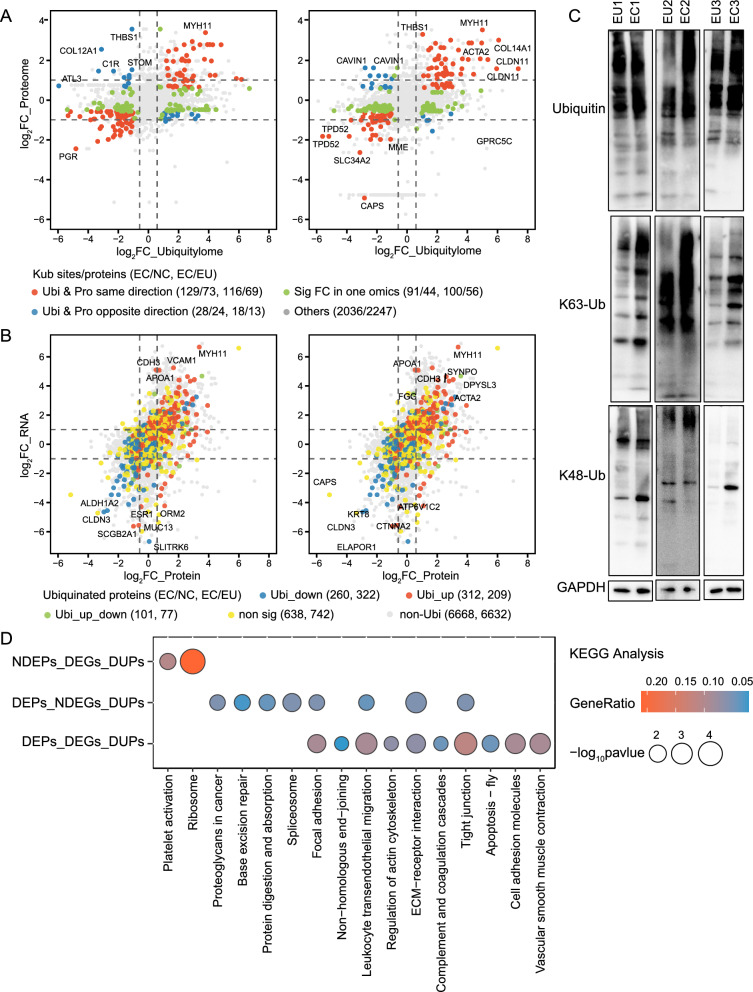
The relationships among transcriptomics, proteomics, and ubiquitylomics in endometriosis. **A** Scatterplot showing the relationship between protein and ubiquitination changes. The log_2_FC in ubiquitination versus protein abundance is plotted, segmented by a two-fold cutoff (p-value < 0.05 and |log_2_FC|> 1 for ubiquitination; p-value < 0.05 and |log_2_FC|> 0.58 for protein). Red dots indicate molecules with aligned trends in ubiquitination and protein changes; blue dots show opposite trends, and green dots represent differences in either ubiquitylomics or proteomics, but not both. **B** Scatterplot illustrating the relationship between changes in protein and mRNA abundances; the color of the dots indicates the classification of ubiquitination status; **C** Utilizing western blot to assess the expression of total ubiquitin, K63-Ub, and K48-Ub proteins in paired EU and EC tissues. **D** Bubble charts showing the enrichment analysis of KEGG pathways for DUPs. Bubble size represents Gene Ratio, and color indicates the − log_10_(p-value) of enrichment. 'DEPs_DEGs_DUPs' denotes molecules with differential expression in all three-omics levels; 'DEPs_NDEGs_DUPs' indicates molecules with no transcriptomic differences but variations in both proteomics and ubiquitylomics; ‘NDEPs_DEGs_DUPs’ refers to molecules with no protein-level differences but variations in both transcriptomics and ubiquitylomics. log_2_FC: log_2_Foldchange; K63-Ub: lysine 63-ubiquitin; K48-Ub: lysine 48-ubiquitin; DUPs: differentially ubiquitinated proteins

We further investigated the role of ubiquitination in regulating protein expression in endometriosis. Scatterplot analysis revealed that 11.8% (73) of ubiquitinated proteins in the EC vs. NC comparison showed consistent trends in protein expression and ubiquitination (red), while only 3.9% (24) exhibited significant opposite changes (blue) (Fig. 5A). Similarly, in the EC vs. EU comparison, 10.6% (69) of proteins displayed consistent trends, while 2.0% (13) showed opposite changes. Integration of ubiquitylome, proteome, and transcriptome data identified a subset of proteins with consistent trends across all three omics levels (Fig. 5B) (Table S7). Additionally, total ubiquitin, K63-Ub, and K48-Ub protein expression levels were found to be increased in EC tissues compared to paired EU tissues (Fig. 5C). These findings suggest that protein expression levels are positively regulated by ubiquitination in ectopic tissues, potentially indicating an increase in non-degradative ubiquitination in endometriosis.

Functional enrichment analysis of different types of DUPs from three omics levels revealed significant enrichment in GO terms (Fig. S5C) and KEGG pathways (Fig. 5D) related to contractile fiber (e.g., ACTA2, ANK3, CALD1, CSRP1, FHL2, FHL3, KRT19, MYH11, MYL9, SPTBN1, SYNPO, TPM1), ECM (e.g., COL1A2, COL6A2, COL6A3, DAG1, HSPG2, ITGA6), cell adhesion (e.g., CLDN3, CLDN10, ITGA6, TNXB, VCAM1), and focal adhesion (e.g., AHNAK, ANXA6, CSRP1, DAG1, FHL2, FHL3, GAK, HSPG2, ILK, ITGA6, MPRIP, MSN, MYH9, PLEC, RPS10, SNTB2, TNS1, VCL). This suggests that ubiquitination of the aforementioned proteins plays a key role in regulating the expression of functional proteins associated with these terms and pathways.

### Multi-omics integration stressed ubiquitination's role in endometriosis fibrosis

**Fig. 6:**
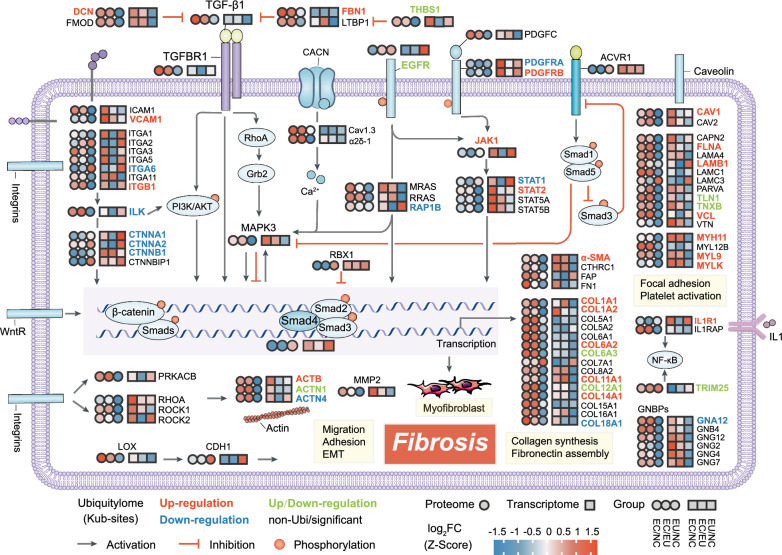
Ubiquitination of key proteins in fibrosis-related pathway in endometriosis. The circle represents proteomics, and the square signifies transcriptomics. The color of the shapes corresponds to the log_2_FC values derived from the differential analysis at each omics level: red denotes upregulation, while blue indicates downregulation. The color-coded depiction of protein symbols reflects ubiquitylomics: red denotes up-regulated Kub-sites on the protein, blue denotes down-regulated Kub-sites, green indicates proteins with both increased and decreased Kub-sites, and black represents proteins lacking Kub-sites or with Kub-sites that lack statistically significant differences. log_2_FC: log_2_Foldchange; Kub: ubiquitinated lysine

Fibrosis is a common feature in all type of endometriosis, contributing to the typical symptoms of pain and infertility. Our findings indicate significant activation of fibrosis-related terms and pathways at both transcriptomic and proteomic levels. Moreover, ubiquitination was identified as a regulator of fibrosis-related molecular functions. Thus, we conducted a systematic analysis of ubiquitination in fibrosis-related proteins. The correlation coefficients of all ubiquitinated proteins associated with fibrosis in the EC/NC and EC/EU groups were 0.32 and 0.36, respectively (Fig. S6A). These results suggest that ubiquitination positively regulates fibrosis-related protein expression levels in ectopic lesions. Analysis of the three omics datasets revealed that 7.9% (41) and 7.2% (37) of fibrosis-related proteins in the EC group had up-regulated Kub-sites compared to the NC and EU groups, respectively. Additionally, 3.3% (17) and 3.9% (20) of proteins had down-regulated Kub-sites, while 2.5% (13) and 2.3% (12) had both up-regulated and down-regulated Kub-sites (Fig. S6B) (Table S8). Lastly, we depicted the ubiquitination of 41 key proteins involved in the fibrosis-related pathway of endometriosis (Fig. 6), with detailed Kub-sites listed in Table S9. These proteins, mostly up-regulated in the EC group, are implicated in the regulation of myofibroblasts, collagen synthesis, fibronectin assembly, focal adhesion, platelet activation, EMT, migration, and adhesion, ultimately contributing to fibrosis in endometriosis.

### Reduced expression of TRIM33 promotes fibrosis in endometriosis

**Fig. 7:**
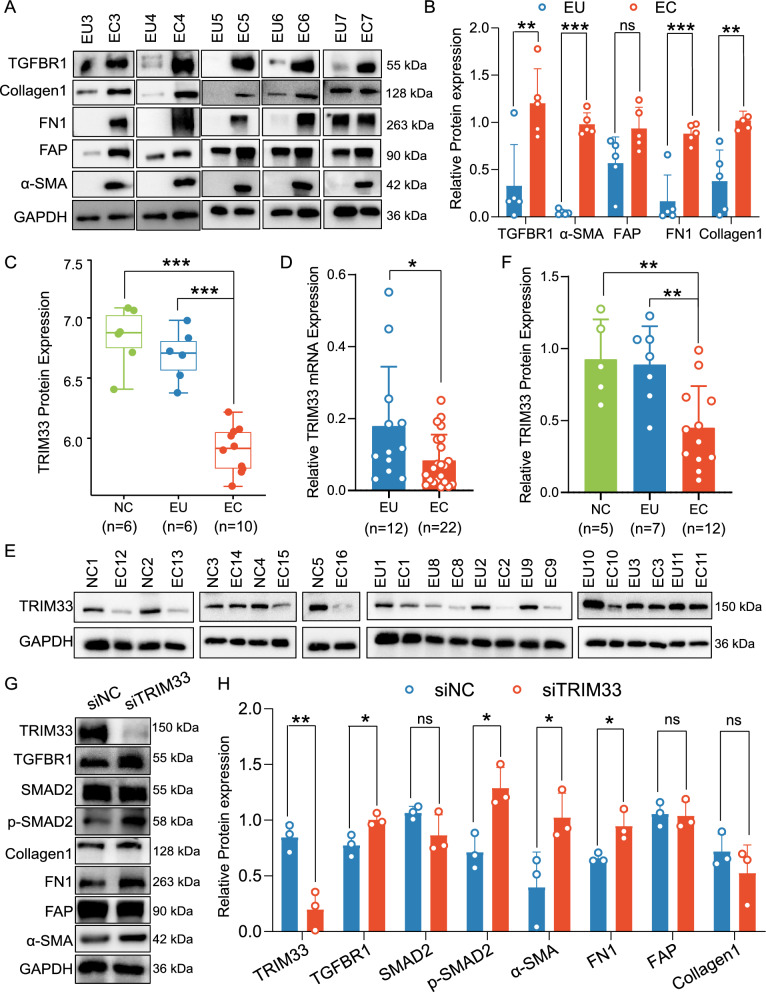
Reduced expression of TRIM33 promotes fibrosis in endometriosis. **A**, **B** Western blot and densitometric analysis of TGFBR1, α-SMA, FAP, FN1, and Collagen1 in paired EU and EC tissues; Data are presented as the mean ± SD (n = 5 in each group). **C** Boxplot illustrating the expression levels of TRIM33 in the NC (n = 6), EU (n = 6), and EC (n = 10) groups as analyzed in the proteomic data. **D** qRT-PCR analysis of TRIM33 mRNA expression in EU (n = 12) and EC (n = 22) tissues; Data are presented as the mean ± SD. **E**, **F** Western blot and densitometric analysis of TRIM33 protein expression in the NC (n = 5), EU (n = 7), and EC (n = 12) tissues; Data are presented as the mean ± SD. **G**, **H** Western blot and densitometric analysis of TRIM33, TGFBR1, SMAD2, p-SMAD2, α-SMA, FAP, FN1, and Collagen1 protein expression in the siNC and siTRIM33 transfected hESCs. Data are presented as the mean ± SD (n = 3 in each group). P values were calculated using unpaired two tailed Student’s t test. *P < 0.05, **P < 0.01, and ***P < 0.001. NC: normal endometria; EU: eutopic endometria; EC: ectopic endometria; siNC: negative control siRNA; siTRIM33: TRIM33 siRNA

Transcriptomic and proteomic analyses both suggest elevated expression of fibrosis-related proteins in endometriosis. Consequently, we conducted western blot on clinical patient tissues to validate this finding. The results indicate an up-regulation of protein expression for TGFBR1/α-SMA/FAP/FN1/Collagen1 in EC tissues compared to paired EU tissues (Fig. 7A, B). However, FAP exhibited no statistically significant difference, potentially due to individual heterogeneity.

Proteomic data indicated a significant downregulation of the E3 ubiquitin ligase TRIM33 protein level in the EC group compared to both the NC and EU groups (Fig. 7C). Thus, we further validated TRIM33 mRNA and protein expression in clinical patient tissues through qPCR and western blot, revealing a significant decrease in TRIM33 levels in the EC group (Fig. 7D–F). These findings suggested a potential association between aberrant TRIM33 expression in endometriotic tissues and the pathology of endometriosis. Prior research has highlighted the significant role of TRIM33 in regulating diseases associated with fibrosis. However, its involvement in the fibrotic progression of endometriosis remains unclear. To investigate this further, we performed transfection of hESCs using TRIM33 siRNA in vitro and assessed its impact on fibrotic protein expression. The results showed that knockdown of TRIM33 in hESCs promoted TGFBR1/p-SMAD2/α-SMA/FN1 protein expressions but did not significantly affect Collagen1/FAP levels (Fig. 7G–H). Consequently, we hypothesize that reduced TRIM33 expression in endometriotic tissues may contribute to the development of endometriosis fibrosis by enhancing TGFBR1/p-SMAD2/α-SMA/FN1 expression.

## Discussion

Endometriosis is a benign gynecological condition with a rising incidence. Fibrosis is present in all pathological types, causing symptoms like dysmenorrhea and infertility, significantly impacting the health of reproductive-age women. Ubiquitination is a necessary PTM regulating various biological processes in human diseases, but its role in endometriosis and associated fibrosis remains unclear. Here, we employed comprehensive multi-omics approaches on two cohorts of endometriosis patients with 39 samples, focusing on transcriptomics, proteomics, and ubiquitylomics profiles. Proteomics identified 8032 unique proteins across all tissues in each group, with 2390 and 1759 proteins exhibiting significant changes in the EC group compared to the NC and EU groups, respectively. Integration of proteomics and transcriptomics revealed genes with concurrent mRNA and protein level changes associated with increased ECM and reduced reproductive functions. Ubiquitylomics detected 8407 ubiquitinated Kub-peptides and 2678 Kub-proteins across tissues, with 1647 and 1698 Kub-sites showing changes in EC compared to NC and EU groups, respectively. Multi-omics integration emphasized the crucial role of ubiquitination in key fibrosis regulators and its positive regulation of fibrosis-related protein expression in ectopic lesions. Further, we identified ubiquitination in 41 pivotal proteins within the fibrosis-related pathway of endometriosis. Finally, we demonstrated TRIM33 downregulation in endometriotic tissues across independent clinical samples, along with its inhibitory effect on fibrosis in hESCs in vitro.

Translated proteins are direct determinants of health and disease. In-depth exploration of disease proteomics is particularly crucial for studying biomarkers and the pathogenesis. Although molecular changes in endometriosis have been studied previously, and several researchers have examined the proteome of endometriosis and characterized disease-related DEPs and pathways or engaged in biomarker discovery [39–41], these did not focus specifically on the process of gene expression in the development of endometriosis. Furthermore, the limitations associated with early mass spectrometry prevented previous studies from revealing the potential regulatory mechanisms of protein expression in the pathogenesis of endometriosis [42, 43]. Here, utilizing advanced mass spectrometry, we generated a proteomic profile of endometriosis with the identification of 8032 unique proteins, concurrently sequencing the transcriptome of the same batch of samples. In agreement with the findings of previous reports, our study confirmed previously reported transcriptomic and proteomic changes in endometriosis, such as an increase in inflammation [12, 44], the immune environment [10–12], and the ECM [10, 12, 37, 38, 45], along with a reduction in reproduction [46–49] related to function and pathways. Endometrial tissue refluxes into the pelvic cavity, where it implants and grows, giving rise to endometriotic lesions. These lesions experience recurring cycles of bleeding and repair, involving immune cell infiltration, release of inflammatory regulators and cytokines, and exposure to a high estrogen environment [2]. These factors lead to abnormal ECM accumulation, causing tissue fibrosis, pelvic adhesions, and impairing fallopian tubes and ovaries, ultimately resulting in reduced reproductive function [50]. The aforementioned pathological changes are considered the classical pathogenic mechanism of endometriosis. Additionally, we also noticed that the previously identified candidate markers, including AOC3 [44], CNN1 [37], EMILIN1 [37], ICAM1 [38], FST [38], PAEP [38], VIM [43], and PRDX6 [43] were aberrantly up-regulated proteins in ectopic endometria. The above results highlight the authenticity and extensive coverage of our proteomic data. Furthermore, the transcriptomic and proteomic data from the same cohort offer valuable insights into the role of PTMs in endometriosis.

After phosphorylation, ubiquitination emerges as the second most abundant and critical PTM. It serves as a terminal regulator in gene expression, impacting crucial cellular processes such as protein stability, localization, activity, interactions, cell signaling, DNA repair, cell cycle, and immune response [51–54]. The ubiquitin expression notably surpasses that in normal endometrium, particularly in ectopic endometrial tissue, correlating with the tissue’s survival [26]. Investigations have identified significant dysregulation of E3 ubiquitin ligases (CHIP, MDM2, TRIM24, TRIM59, TRIM65, SMURF1, ITCH) and DUBs (UCHL1, USP10) in ectopic lesions [27–35]. These enzymes influence the degradation of downstream substrates, impacting estrogen regulation, inflammation, proliferation, migration, invasion of endometrial stromal cells, and glycolysis, thereby affecting the progression of endometriosis. Notably, TRIM59 in ectopic lesions, through ubiquitination, regulates PPM1A degradation, activating the TGF-β/SMAD2/3 pathway and thus promoting fibrosis in endometriosis [30]. The findings from these studies indicate diverse alterations in protein ubiquitination and the expression of modifying enzymes in ectopic lesions. This imbalance in ubiquitination homeostasis during endometriosis pathogenesis disrupts normal protein homeostasis, crucial for the onset and progression of the condition. Despite the limited research on ubiquitination in the pathological mechanisms of endometriosis, confirmation of the overall protein ubiquitination homeostasis in ectopic lesions remains pending. Further exploration is also needed to understand the key enzymes responsible for ubiquitination abnormalities. In this study, we identified 266 DUBs and UBs across all tissues within the proteomics data and distinguished 52 and 38 dysregulated enzymes, respectively, in the EC group. Among these, the altered protein expressions E3 ubiquitin ligases (TRIM24, TRIM65) and DUBs (UCHL1, USP10) were consistent with previous studies. This dysregulation of DUBs and UBs in the EC group suggests abnormal ubiquitination profiles in ectopic lesions. Further ubiquitylomics detected 8407 Kub-peptides and 2678 Kub-proteins across tissues, with 1647 and 1698 Kub-sites showing changes in EC compared to NC and EU groups, respectively. These DUPs were linked to processes like platelet aggregation, cell adhesion, cytoskeleton, contractile fiber, and signaling pathways. These changes in crucial pathways influenced by ubiquitination may play a role in endometriosis development. In summary, we have, for the first time, delineated a profile of proteins regulated by ubiquitination in endometriosis, highlighting potential functional changes they might influence.

Ubiquitination plays a role in regulating protein expression, localization, and interactions, employing various linkage modes. K48-modification is well-studied for its role in protein degradation, while K63-modification regulates protein interactions, influencing the activation of signaling pathways [55, 56]. To understand how ubiquitination influences proteins in endometriosis, we explored the relationship between the global proteome and ubiquitylome. Correlation coefficients analysis based on log_2_FC that the values of all ubiquitinated proteins in the EC/NC and EC/EU groups had correlation coefficients of 0.22 and 0.32, respectively, while the coefficient for the EU/NC group was − 0.17. This pattern was more evident in DUPs, with coefficients of 0.42 (EC/NC), 0.52 (EC/EU), and − 0.32 (EU/NC). Besides, in the EC/NC and EC/EU groups, 11.8% (73) and 10.6% (69) of ubiquitinated proteins, respectively, exhibited consistent trends in both protein expression and ubiquitination changes. Conversely, only 3.9% (24) and 2.0% (13) demonstrated significantly opposite changes. These results indicated that, compared to the NC and EU groups, the overall proteome and ubiquitylome in the EC group showed a positive correlation, suggesting a positive regulatory impact of ubiquitination on protein expression in ectopic lesions. We investigated the expression of two common ubiquitin types, K48 and K63, in endometriosis and observed an upregulation in their protein expression in ectopic tissues. Therefore, we postulate that the prevalent form of ubiquitination in ectopic tissues of endometriosis is non-degradative ubiquitination, which warrants attention and validation in future research.

The comprehensive analysis of DUPs across three omics levels revealed significant enrichment in pathways related to contractile fiber, ECM, cell adhesion, and focal adhesion, suggesting ubiquitination emerged as a critical regulator of fibrosis-related molecular functions. Furthermore, our systematic analysis indicated a positive correlation between ubiquitination and fibrosis-related protein expression in ectopic lesions. Additionally, we observed distinct ubiquitination in fibrosis-related proteins, with a notable proportion showing up-regulation in endometriosis.

Fibrosis in endometriosis is known to involve activated platelets, macrophages, and myofibroblasts, ultimately promoting elevated levels of TGF-β and collagen deposition [50]. Activated myofibroblasts in endometriotic lesions are identified by the presence of alpha-smooth muscle actin (α-SMA). Immunostaining with α-SMA antibodies yields positive results in all endometriotic lesions [57–59]. Once activated, these myofibroblasts increase their proliferation, migration, and production of cytokines and interstitial matrix [6]. Continuous myofibroblast activity results in the accumulation and contraction of collagen ECM. In our omics results, we observed an elevation in the expression of critical fibrosis-associated proteins, including TGFBR1, α-SMA, CTHRC1, FAP, FN1, and 15 collagen proteins, in endometriosis. Meanwhile, we validated the heightened expression of TGFBR1/α-SMA/FAP/FN1/Collagen1 proteins in EC tissues through western blot in independent clinical samples, indicating an exacerbation of fibrosis in endometriotic tissues. More importantly, Kub-sites were differentially upregulated in proteins such as α-SMA, COL1A1/2, COL6A2, COL11A1, and COL14A1 in ectopic tissues. COL6A3 and COL12A1 exhibited both upregulated and downregulated Kub-sites, whereas only COL18A1 showed downregulated Kub-sites. As commonly understood, collagen undergoes degradation primarily through collagenases and matrix metalloproteinases (MMPs) [60]. Therefore, we hypothesized that ubiquitination may exert a non-degradative regulatory role in the mentioned fibrosis-promoting proteins, potentially influencing transcription, protein localization and interactions. For example, TRAF6 mediates KLF5 K63-linked ubiquitination, promoting nuclear localization and enhancing gene activation, contributing to renal fibrosis regulation [61]. Non-degradative ubiquitination fine-tunes GTPase function by modifying protein interaction network [62]. Recent studies also indicate a positive correlation between the observed increase in K63 ubiquitination levels, both in vivo and in vitro, and the expression of α-SMA [63]. Inhibiting Lysine 63 ubiquitination can hinder the progression of renal fibrosis in vivo [64].

The investigation into platelet activation in endometriosis has not been thoroughly documented. The available research suggested that platelets played a vital role in endometriosis-related fibrosis by releasing significant amounts of growth factors, cytokines, and chemokines (e.g., TGF-β1, PDGF, EGF, CTGF) when activated within the lesion [50]. During activation, platelet integrins interact with specific ECM components, forming focal adhesions. This process triggers cytoskeletal rearrangements crucial for focal adhesion formation and stability during ECM adhesion. This microenvironment supports processes like epithelial-to-mesenchymal transition (EMT), fibroblast-to-myofibroblast transition (FMT), smooth muscle metaplasia (SMM), and endothelial-to-mesenchymal transition (EndoMT), potentially directly promoting fibrosis [65]. In our study, we observed increased levels of several proteins in ectopic tissues, including those related to focal adhesion and platelet activation (e.g., CAPN2, FLNA, Laminin, PARVA, TLN1, TNXB, VCL, VTN, MYH11, MYL12B, MYL9, MYLK), proteins involved in cytoskeleton formation (ACTB, ACTN1, ACTN4), and integrin family members (ITGA1, ITGA2, ITGA3, ITGA5, ITGA11, ITGB1). Moreover, proteins closely linked to these processes, including RHOA/ROCK signaling pathway proteins, play a role in controlling cell migration and invasion, showing increased expression. Our prior studies have reported that the abnormal activation of the RhoA/ROCK pathway in endometriosis contributes to the development of the condition by activating the estrogen/ERα/ERK pathway, resulting in EMT and enhanced cell proliferation [66]. Among the mentioned proteins, differential Kub-sites were observed in FLNA, LAMB1, TLN1, TNXB, VCL, MYH11, MYL9, MYLK, ACTB, ACTN1, ACTN4, and ITGB1.

TGF-β stands out as a pivotal pro-fibrotic cytokine that triggers fibrosis by activating the classical signaling pathway, fostering the formation of the Smad2/3/4 complex, and inducing the transcription of pro-fibrotic molecules. This cascade culminates in the activation of myofibroblasts, facilitating matrix deposition and promoting fibrosis. Concurrently, the intricate interplay between TGF-β and various signaling pathways, including RhoA/ROCK, PI3K/AKT, EGFR, ERK, RAS, JAK/STAT, and Wnt/β-catenin, significantly influences the progression of tissue fibrosis [67]. Studies suggest a pivotal role for TGF-β in the initiation and progression of fibrosis in endometriosis [68]. Endometrial cells within endometriotic lesions can synthesize TGF-β1, accumulate in the surrounding ovarian tissue, disrupt ECM, and contribute to fibrosis around endometriotic cysts. Activation of TGF-β1/Smad, PI3K/AKT, MAPK/ERK, and Wnt/β-catenin pathways has been linked to the survival, proliferation, migration, and collagen contraction of interstitial cells in endometriosis [69]. Our investigation additionally unveiled the ubiquitination of key proteins in these signaling pathways in endometriosis. For example, elevated Kub-sites were observed in PDGFRB, JAK1, and STAT2 proteins in ectopic tissue, while PDGFRA, ILK, RAP1B, and catenin exhibited down-regulated Kub-sites. Simultaneously, EGFR displayed both up-regulated and down-regulated Kub-sites. The well-established role of ubiquitination in regulating EGFR influences its functions in cell signaling, endocytosis, stability, and degradation [70, 71]. However, as of now, ubiquitination modification of EGFR in endometriosis remains unreported. In conclusion, the complex interplay between TGF-β and various signaling pathways, along with the identification of ubiquitination modifications in key proteins, offers new insights into the molecular mechanisms of tissue fibrosis in endometriosis. These findings deepen our understanding of the regulatory networks governing fibrotic processes and open avenues for targeted therapeutic interventions in endometriosis-related fibrosis.

It is widely acknowledged that ubiquitination is a dynamic balancing process, with E3 ubiquitin ligases tasked with specifically recognizing and attaching ubiquitin to substrate proteins, while DUBs can dynamically remove ubiquitination modifications from substrate proteins [23]. Given the pronounced abnormalities in the expression and ubiquitination of fibrosis-related proteins as indicated by transcriptomics, proteomics, and ubiquitylomics, and we systematically uncovered differences in UBs and DUBs in endometriosis. Hence, we endeavored to investigate whether aberrantly expressed E3 and DUBs play a role in regulating fibrosis in endometriosis. Among the 28 differentially regulated enzymes identified in EC/NC and EC/EU comparisons, we honed in on three members of the transcriptional intermediary factor 1 (TIF1) family, alongside α/TRIM24, β/TRIM28, and γ/TRIM33 [72], whose protein expressions were notably downregulated in endometriotic tissues, indicating their potential involvement in the pathological processes of endometriosis. Therein, TRIM33, comprising (ring-box-coiled-coil) RBCC, Middle linker, PHD, and Bromo domains, functions as both an E3 ubiquitin-ligase and a transcriptional co-regulator. It plays a role in embryonic development, hematopoiesis, fibrosis, metabolism, and regulation associated with tumors [73]. Notably, TRIM33 has been shown to act as a negative regulator of the TGF-β1 signaling pathway, exerting its effects through SMAD4 ubiquitination and turnover of TGF-β receptors [74–76]. However, the abnormal dysregulation of TRIM33 in endometriosis and its role in regulating endometriosis-associated fibrosis remain unclear. Our study further confirmed the downregulation of TRIM33 in independent clinical ectopic tissues via western blot, which is consistent with the omics results. Additionally, we utilized siRNA to silence TRIM33 expression in hESCs cells to investigate its potential impact on fibrosis-related protein levels. The results revealed that knockdown of TRIM33 significantly enhanced the protein expression of TGFBR1/p-SMAD2/α-SMA/FN1, suggesting that the reduced levels of TRIM33 protein in ectopic tissues may exacerbate endometriosis-associated fibrosis. Future research should concentrate on elucidating the in vivo functions of TRIM33 and its specific molecular mechanisms affecting fibrosis-related proteins.

Overall, the present study, employing multi-omics approaches, presents a novel perspective for the first time on the ubiquitination profile and the aberrant expression of TRIM33 in endometriosis, highlighting their pivotal roles in fibrosis and their potential as future therapeutic targets.

## Conclusions

In this study, we comprehensively profiled the ubiquitination landscape of endometriosis for the first time. Through proteomics, transcriptomics, and ubiquitylomics analyses of endometrial tissues from two patient cohorts, we uncovered significant dysregulation of ubiquitin-related enzymes and ubiquitinated proteins. By comparing tissues in pathological and healthy states, we delineated ubiquitinated proteins associated with the etiology of endometriosis and revealed their regulatory roles in relevant biological processes. We observed a pivotal role of ubiquitination in the fibrotic process, particularly in processes related to the activity of myofibroblasts and collagen deposition in ectopic tissues. Besides, we uncovered the downregulation of the E3 ubiquitin ligase TRIM33 in endometriotic tissues, along with its negative regulatory effect on fibrosis-related proteins TGFBR1/p-SMAD2/α-SMA/FN1 in vitro. It is important to note that, while our study provides compelling evidence supporting the crucial role of ubiquitination in endometriosis and the negative regulatory effect of TRIM33 on fibrosis, the specific regulatory mechanisms require further in-depth functional studies. This study contributes to a deeper understanding of endometriosis pathogenesis and highlights the potential of targeting ubiquitination pathways for therapeutic intervention in this debilitating condition.

### Supplementary Information

**Additional file 1. Fig. S1** Functional enrichment analysis of transcriptomic data in endometriosis. **Fig. S2** Functional enrichment analysis of proteomics data in endometriosis. **Fig. S3** Functional enrichment analysis of different types of DEPs and DEGs. **Fig. S4** Ubiquitination profiling landscape in endometriosis. **Fig. S5** The relationships among transcriptomics, proteomics, and ubiquitylomics in endometriosis. **Fig. S6** The relationship among fibrosis-related proteins across three omics datasets.

**Additional file 2.** Clinical information for the sequencing and validation cohorts (Table S1)

**Additional file 3.** Enriched results of GO and KEGG analysis for DEGs in transcriptomics (Table S2)

**Additional file 4.** Enriched results of GO and KEGG analysis for DEPs in proteomics (Table S3)

**Additional file 5.** Integrated transcriptome and proteome analysis results (Table S4)

**Additional file 6.** A list of ubiquitination-related regulatory enzymes identified by proteomics (Table S5)

**Additional file 7.** Enriched results of GO and KEGG analysis for DUPs in ubiquitylomics (Table S6)

**Additional file 8.** Integration of ubiquitylome, proteome, and transcriptome data (Table S7)

**Additional file 9.** Results from integrated analysis of three omics datasets for 518 fibrosis-related proteins (Table S8)

**Additional file 10.** Detailed Kub-sites of 41 key proteins in the fibrosis-related pathway of endometriosis (Table S9)

## Data Availability

The proteomic data are deposited in ProteomeXchange Consortium (Project ID: PXD036497) via the iProX repository partner with the dataset identifier IPX0004973000. The transcriptomic data can be found here: Gene Expression Omnibus (GEO) database (GSE212787). The ubiquitylomics data will be made available on request.
