# Supplementary material for: Multi-omics integration highlights the role of ubiquitination in endometriosis fibrosis
Source: J Transl Med. 2024 May 12;22:445. doi: 10.1186/s12967-024-05245-0 (PMC11089738; doi:10.1186/s12967-024-05245-0)
Supplement: Supplementary file 1 — Additional file 1. Fig. S1 Functional enrichment analysis of transcriptomic data in endometriosis. Fig. S2 Functional enrichment analysis of proteomics data in endometriosis. Fig. S3 Functional enrichment analysis of different types of DEPs and DEGs. Fig. S4 Ubiquitination profiling landscape in endometriosis. Fig. S5 The relationships among transcriptomics, proteomics, and ubiquitylomics in endometriosis. Fig. S6 The relationship among fibrosis-related proteins across three omics datasets. [file 12967_2024_5245_MOESM1_ESM.docx]

**Multi-Omics Integration Highlights the Role of Ubiquitination in Endometriosis Fibrosis**

Mengjie Yang ^1, 2^ ^†^, Hong Jiang ^4^ ^†^, Xinyu Ding ^1^, Lu Zhang ^1, 2^, Huaying Zhang ^1^, Jiahao Chen ^1^, Lijun Li ^1^, Qinxin He ^3*^, Zhixiong Huang ^1*^, Qionghua Chen ^1, 2­­­­­*^

^1^ Laboratory of Research and Diagnosis of Gynecological Diseases of Xiamen City, Clinical Medical Research Center for Obstetrics and Gynecology Diseases of Fujian Province, Department of Obstetrics and Gynecology, the First Afﬁliated Hospital of Xiamen University, School of Medicine, Xiamen University, Xiamen, China

^2^ National Institute for Data Science in Health and Medicine, Xiamen University, Xiamen, China

^3^ Department of Obstetrics and Gynecology, the First Affiliated Hospital of Fujian Medical University, Fuzhou, China

^4^ Reproductive Medicine Center, the First Affiliated Hospital of Fujian Medical University, Fuzhou, China

^†^ These authors contributed equally to this work.

*** Correspondence address:**

**Qionghua Chen,** Tel: +86-138-5999-3398, E-mail: [cqhua616@126.com](mailto:cqhua616@126.com);

**Zhixiong Huang,** Tel: +86-139-5010-4214, E-mail: [zhixiong0815@163.com](mailto:zhixiong0815@163.com);

**Qinxin He,** Tel: +86-139-5012-9163, E-mail: hexinqin@fjmu.edu.cn.

**
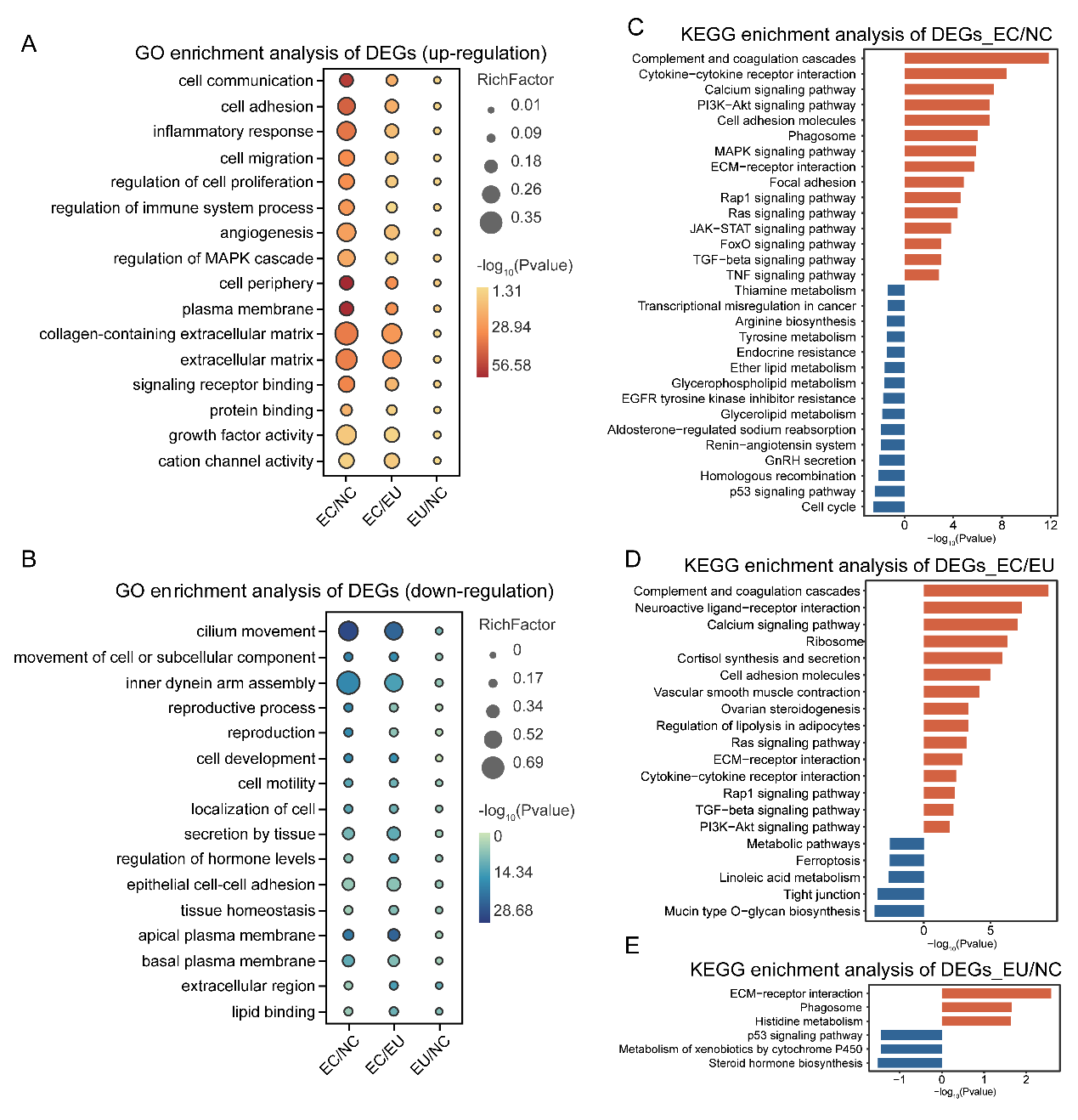
**

**Fig. S1 Functional enrichment analysis of transcriptomic data in endometriosis.** **(A-B)** The bubble chart of up-regulated **(A)** and downregulated **(B)** GO term enrichment analysis of DEGs in EC versus NC, EC versus EU, and EU versus NC groups, respectively. Bubble size indicates a rich factor, and color represents the -log_10_(p-value) of enrichment. **(C-E)** Enrichment of DEGs in EC versus NC **(C)**, EC versus EU **(D)**, and EU versus NC **(E)** groups, respectively, in KEGG pathways. The x-axis represents the relative -log_10_(p-value), the red bar represents up-regulated, and the blue represents downregulated. EC: ectopic endometria; EU: eutopic endometria; NC: normal control endometria; DEGs: differentially expressed genes; log_2_FC: log_2_Foldchange.


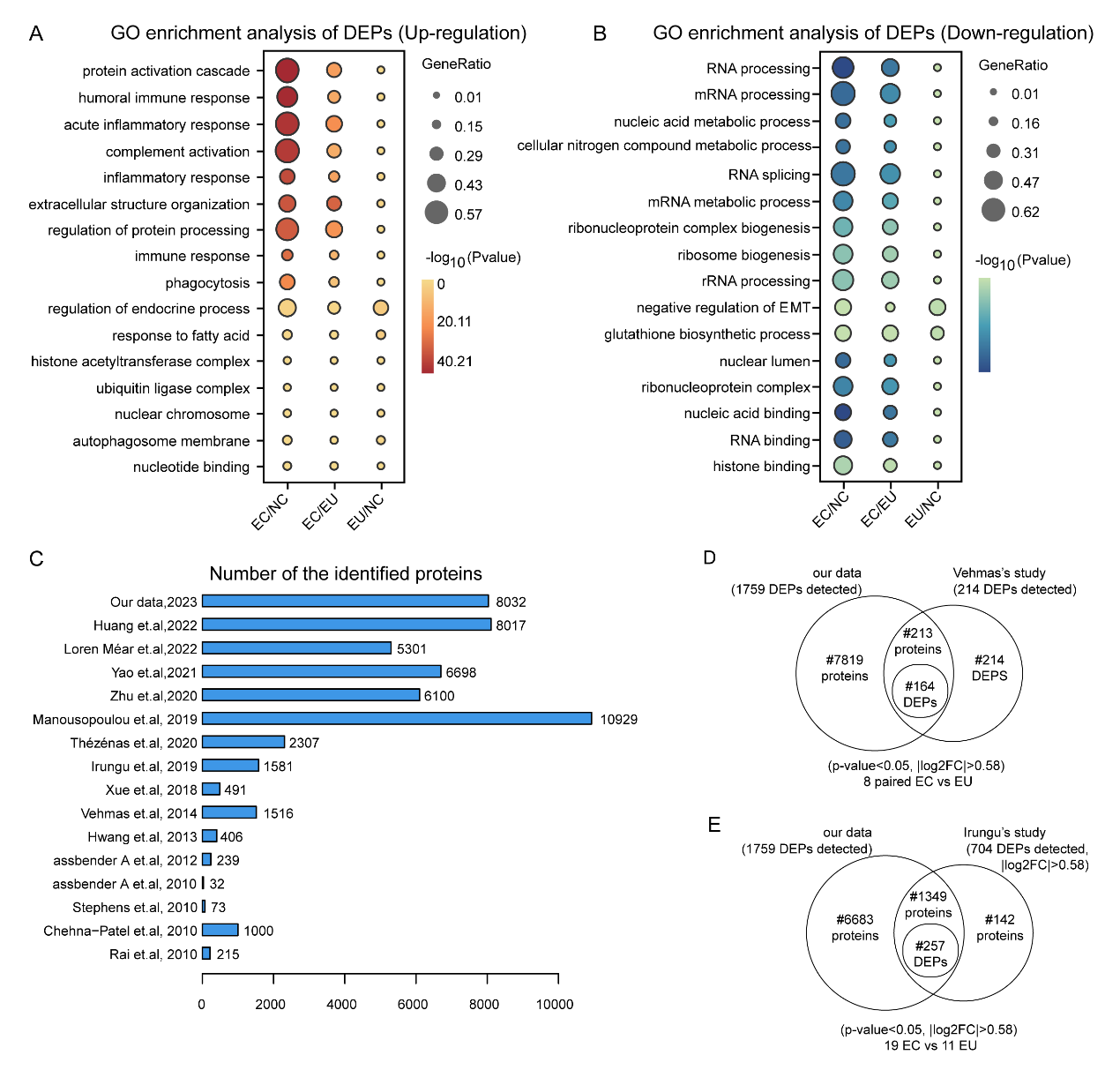


**Fig. S2 Functional enrichment analysis of proteomics data in endometriosis.**

**(A-B)** Bubble charts illustrating the enrichment analysis of GO terms for up-regulated **(A)** and downregulated **(B)** DEPs in EC versus NC, EC versus EU, and EU versus NC groups, respectively. The size of each bubble indicates the Gene Ratio and color represents the -log_10_(p-value) of enrichment. **(C)** We examined recent proteomic research articles analyzing the endometrial proteome in endometriosis and compared the number of identified proteins with our results. **(D-E)** The Venn diagrams illustrate the overlap of human endometriosis-associated proteins from previously conducted proteomic analyses, which identified proteins in both EC and EU endometria with endometriosis, and the DEPs (p-value<0.05, |log_2_FC|>0.58) identified in these studies. DEPs: differentially expressed proteins.


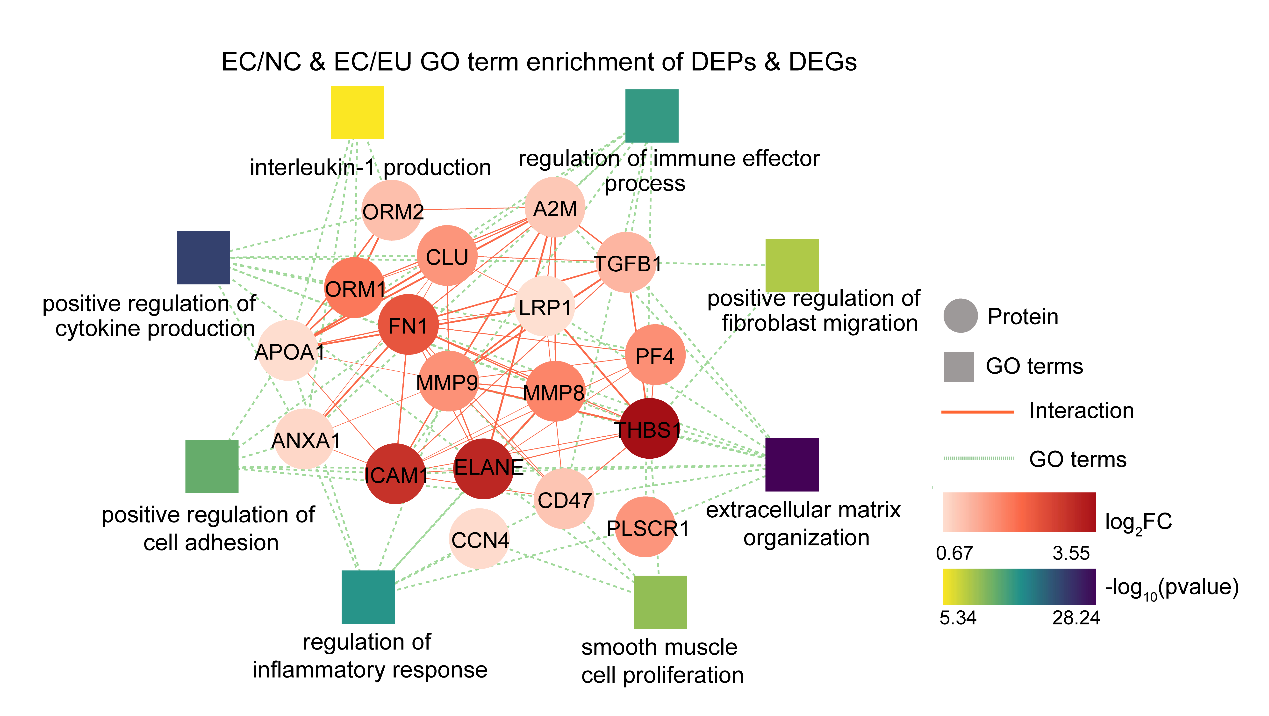


**Fig. S3 Functional enrichment analysis of different types of DEPs and DEGs.** Simplified diagram showing protein interactions in enriched GO terms related to inflammation, immune response, cytokines, and extracellular matrix. Red solid lines indicate protein interactions, green dotted lines represent GO terms. The color bar, ranging from light red to dark red, represents the protein level fold change from decrease to increase (EC versus NC). The significance of pathways is depicted by -log_10_(p-value) with dark purple indicating the highest significance.

**
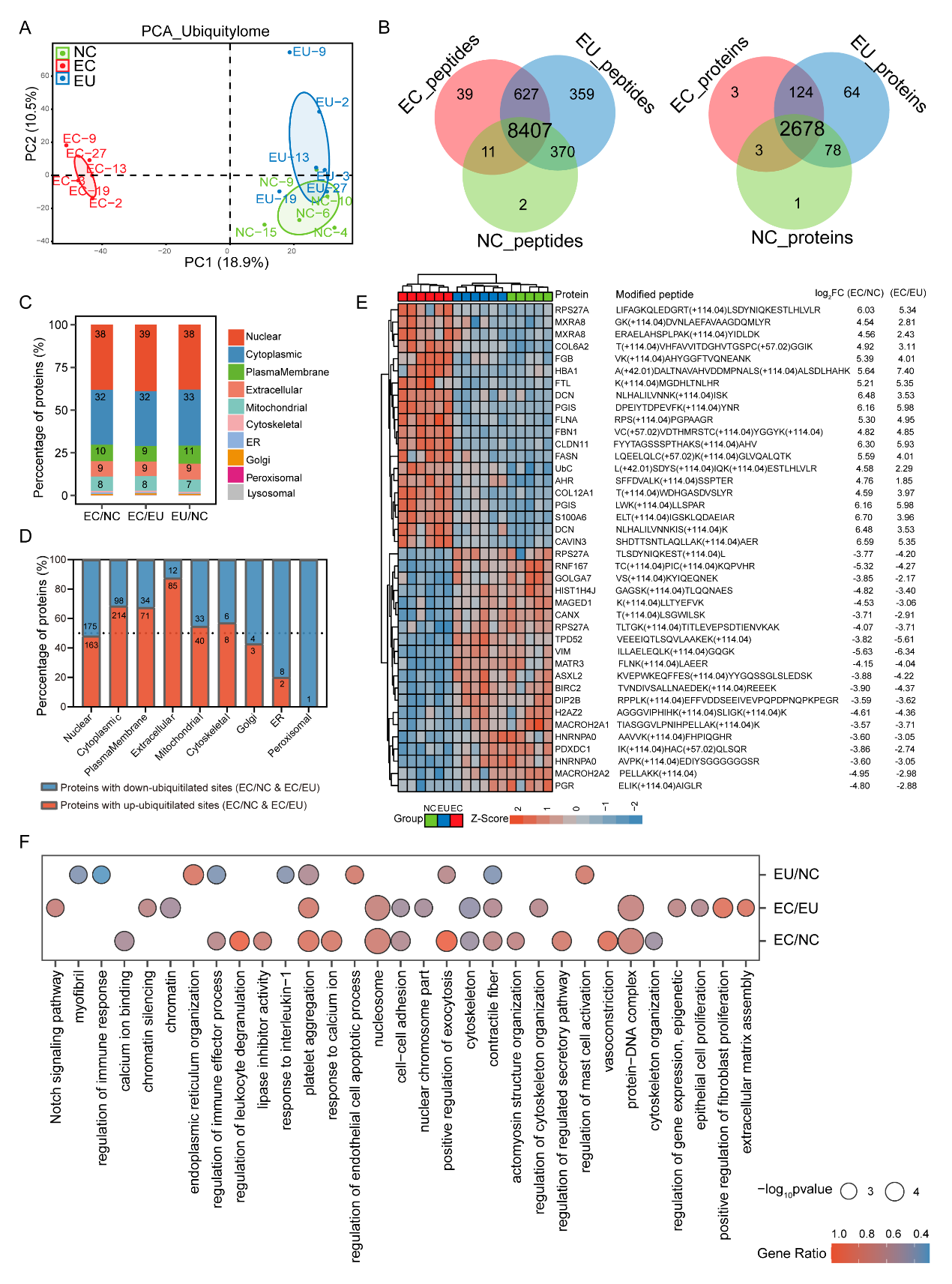
**

**Fig. S4 Ubiquitination profiling landscape in endometriosis. (A)** PCA of ubiquitylomics data, illustrating the separation of EC, EU, and NC samples; **(B)** The Venn diagram illustrates the number of identified peptides and proteins in each group across all samples; **(C-D)** Subcellular localization analysis of DUPs; **(E)** The heatmap displayed the top 20 differentially Kub-sites and corresponding proteins in the EC group compared to the EU and NC groups. **(F)** Bubble charts illustrating the enrichment analysis of GO terms of DUPs in EC versus NC, EC versus EU, and EU versus NC groups, respectively. The size of each bubble indicates the Gene Ratio and color represents the -log_10_(p-value) of enrichment. DUPs: differentially ubiquitinated proteins. NC: normal endometria; EU: eutopic endometria; EC: ectopic endometria; Kub: ubiquitinated lysine.


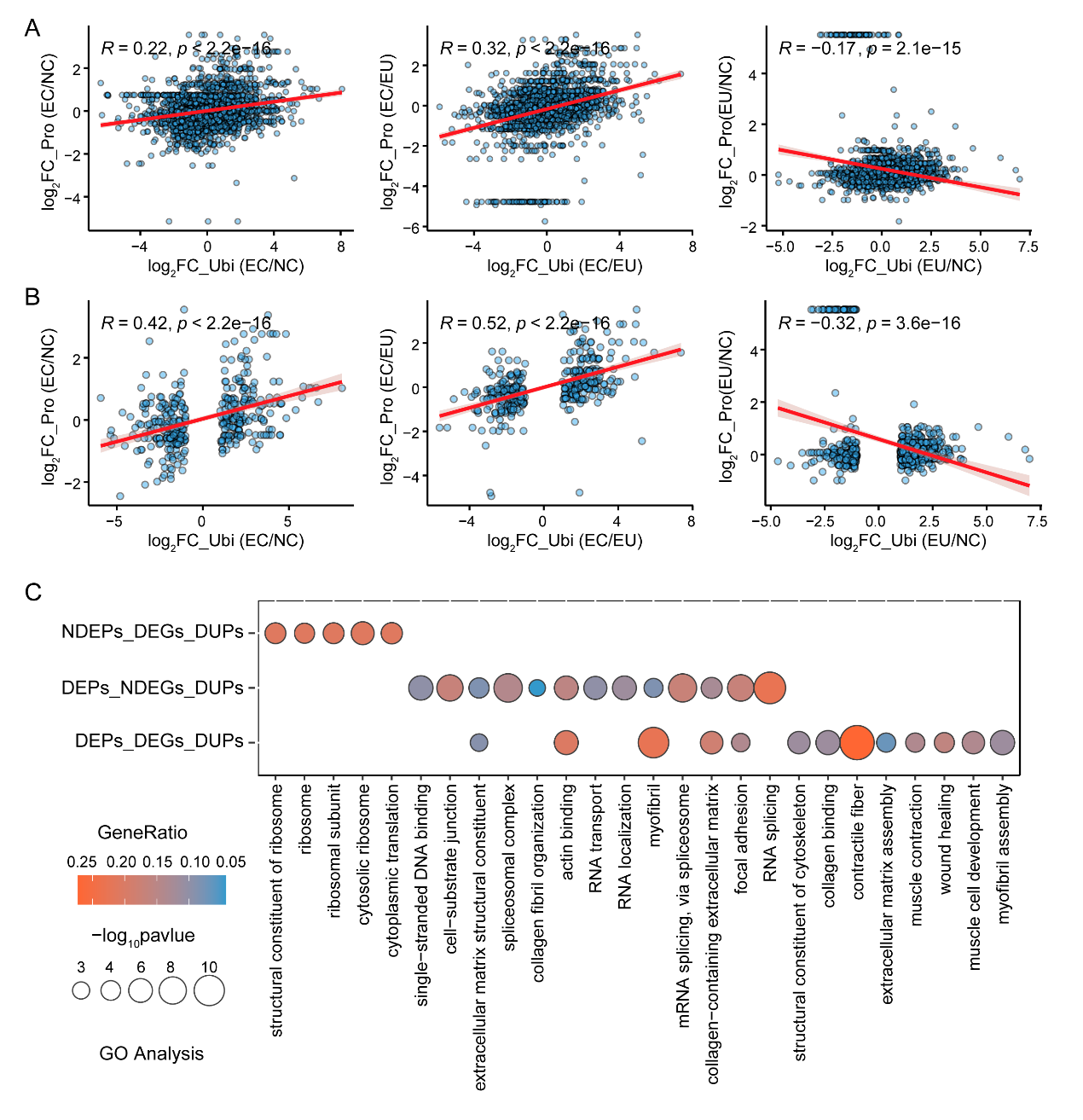


**Fig. S5 The relationships among transcriptomics, proteomics, and ubiquitylomics in endometriosis. (A)** Correlation of FC between the proteome and ubiquitylome for all ubiquitinated protein pairs; **(B)** Correlation of fold changes between the proteome and ubiquitylome for significant ubiquitinated protein pairs; **(C)** Bubble charts showing the enrichment analysis of GO terms for DUPs. Bubble size represents Gene Ratio, and color indicates the -log_10_(p-value) of enrichment. 'DEPs_DEGs_DUPs' denotes molecules with differential expression in all three-omics levels; 'DEPs_NDEGs_DUPs' indicates molecules with no transcriptomic differences but variations in both proteomics and ubiquitylomics; 'NDEPs_DEGs_DUPs' refers to molecules with no protein-level differences but variations in both transcriptomics and ubiquitylomics. DUPs: differentially ubiquitinated proteins.


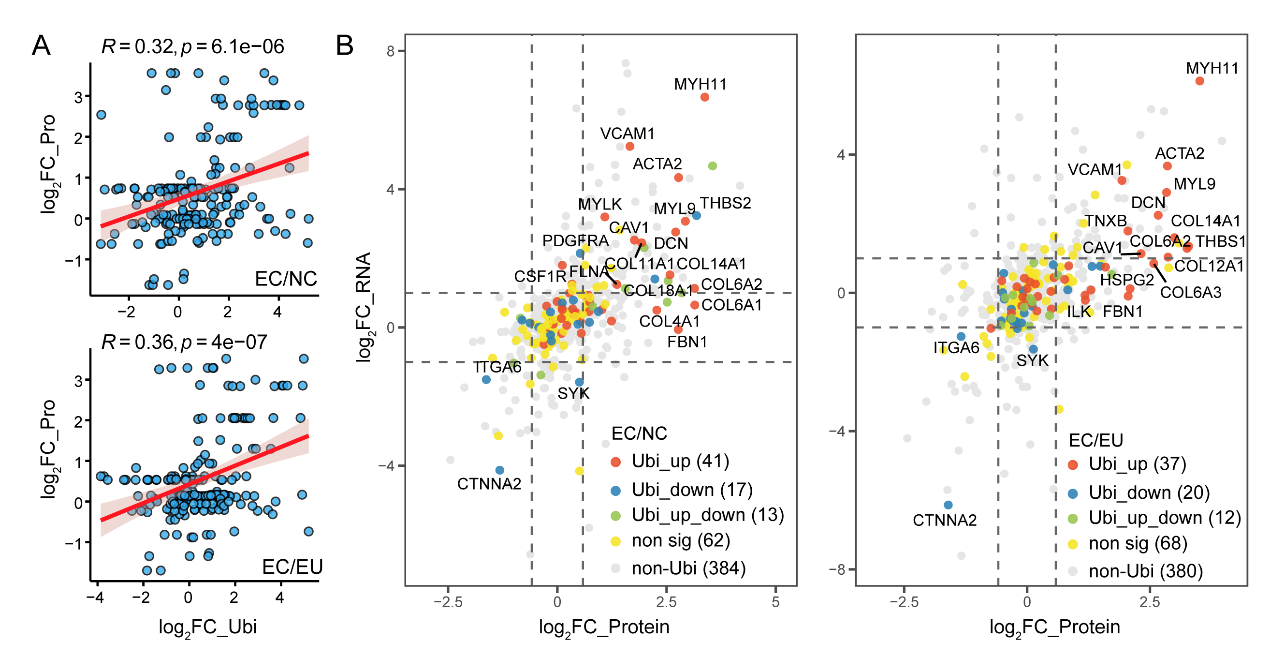


**Fig. S6 The relationship among fibrosis-related proteins across three omics datasets.** **(A)** FC-based correlation between fibrosis-associated proteins undergoing ubiquitination in the proteomics and ubiquitylomics datasets; **(B)** Scatterplot illustrating the relationship between changes in 517 fibrosis-associated proteins and their corresponding mRNA abundances; the color of the dots indicates the classification of ubiquitination status. FC: Foldchange.
