# Supplementary material for: Multi-omics integration highlights the role of ubiquitination in endometriosis fibrosis
Source: J Transl Med. 2024 May 12;22:445. doi: 10.1186/s12967-024-05245-0 (PMC11089738; doi:10.1186/s12967-024-05245-0)
Supplement: Supplementary file 2 — Additional file 2. Clinical information for the sequencing and validation cohorts (Table S1) [file 12967_2024_5245_MOESM2_ESM.docx]

**Table S1. Clinical characteristics of the study population**

| **Characters** |  |  | **Cohort 1** | | | |  | | | **Cohort 2** | | | | | | |  | | **Cohort 3** | | | | | | | | | |
| --- | --- | --- | --- | --- | --- | --- | --- | --- | --- | --- | --- | --- | --- | --- | --- | --- | --- | --- | --- | --- | --- | --- | --- | --- | --- | --- | --- | --- |
|  |  | **Control (n=6)** |  | **Endometriosis (n=10)** |  | | ***P*** | |  | **Control (n=5)** |  | **Endometriosis (n=6)** |  | | ***P*** | |  |  | **Control (n=5)** | |  | | **Endometriosis (n=22)** | |  | | ***P*** | |
| **Age (years)** |  | 32.17 ± 4.49 |  | 32.30 ± 6.69 |  |  | 0.96 | |  | 40.00 ± 6.12 |  | 36.83 ± 6.91 |  |  | 0.63 | |  |  | 32.60 ± 2.40 | |  |  | 34.27 ± 5.82 | |  |  | 0.54 | |
| **BMI (kg/m^2^)** |  | 23.45 ± 4.71 |  | 21.28 ± 2.56 |  |  | 0.25 | |  | 22.43 ± 1.87 |  | 21.44 ± 1.32 |  |  | 0.19 | |  |  | 22.15 ± 6.14 | |  |  | 22.00 ± 2.36 | |  |  | 0.48 | |
| **CA125 (U/mL)** |  | - |  | 56.55 ± 59.31 |  |  |  | |  | - |  | 58.43 ± 27.38 |  |  |  | |  |  | - | |  |  | 103.10 ± 133.70 | |  |  |  | |
| **AMH (ng/mL)** |  | - |  | 2.01 ± 1.49 |  |  |  |  |  | - |  | 2.04 ± 1.62 |  |  |  |  |  |  | - | |  |  | 2.16 ± 1.46 | |  |  |  |  |
| **Dysmenorrhoea (n)** |  | 4 |  | 7 |  |  |  |  |  | 1 |  | 2 |  |  |  |  |  |  | 0 | |  |  | 17 | |  |  |  |  |
| **Reproductive history (n)** |  |  |  |  |  |  |  |  |  |  |  |  |  |  |  |  |  |  |  | |  |  |  | |  |  |  |  |
| N with subfertility |  | 4 |  | 0 |  |  |  |  |  | 1 |  | 1 |  |  |  |  |  |  | 3 | |  |  | 0 | |  |  |  |  |
| N with fertility |  | 2 |  | 7 |  |  |  |  |  | 4 |  | 5 |  |  |  |  |  |  | 2 | |  |  | 17 | |  |  |  |  |
| N with unmarried |  | 0 |  | 3 |  |  |  |  |  | 0 |  | 0 |  |  |  |  |  |  | 0 | |  |  | 5 | |  |  |  |  |
| **Lesions (n)** |  |  |  |  |  |  |  |  |  |  |  |  |  |  |  |  |  |  |  | |  |  |  | |  |  |  |  |
| A unilateral cyst |  | - |  | 6 |  |  |  |  |  | - |  | 4 |  |  |  |  |  |  | - | |  |  | 11 | |  |  |  |  |
| A bilateral cyst |  | - |  | 4 |  |  |  |  |  | - |  | 2 |  |  |  |  |  |  | - | |  |  | 11 | |  |  |  |  |
| N with DIE |  | - |  | 6 |  |  |  |  |  | - |  | 1 |  |  |  |  |  |  | - | |  |  | 15 | |  |  |  |  |
| **ASRM Stage (n)** |  | | | | |  | |  | |  | | | |  | |  | |  | |  | |  | |  | |  | |  |
| I |  | - |  | 1 |  | |  | |  | - |  | - |  | |  | |  | | - | |  | | - | |  | |  | |
| II |  | - |  | 2 |  |  |  |  |  | - |  | - |  |  |  |  |  |  | - | |  |  | - | |  |  |  |  |
| III |  | - |  | 3 |  |  |  |  |  | - |  | - |  |  |  |  |  |  | - | |  |  | 12 | |  |  |  |  |
| IV |  | - |  | 4 |  |  |  |  |  | - |  | 6 |  |  |  |  |  |  | - | |  |  | 10 | |  |  |  |  |

BMI: body mass index; DIE: deep infiltrating endometriosis; ASRM: American Society for Reproductive Medicine.
